# Eomes function is conserved between zebrafish and mouse and controls left-right organiser progenitor gene expression via interlocking feedforward loops

**DOI:** 10.3389/fcell.2022.982477

**Published:** 2022-08-25

**Authors:** Conor D. Talbot, Mark D. Walsh, Stephen J. Cutty, Randa Elsayed, Eirini Vlachaki, Ashley E. E. Bruce, Fiona C. Wardle, Andrew C. Nelson

**Affiliations:** ^1^ School of Life Sciences, Gibbet Hill Campus, University of Warwick, Coventry, United Kingdom; ^2^ Randall Centre for Cell and Molecular Biophysics, New Hunt’s House, Guy’s Campus, King’s College London, London, United Kingdom; ^3^ Warwick Medical School, Gibbet Hill Campus, University of Warwick, Coventry, United Kingdom; ^4^ Department of Cell and Systems Biology, University of Toronto, Toronto, ON, Canada

**Keywords:** *Eomes*, T-box, *vgll4l*, left-right organiser, zebrafish

## Abstract

The T-box family transcription factor Eomesodermin (Eomes) is present in all vertebrates, with many key roles in the developing mammalian embryo and immune system. Homozygous Eomes mutant mouse embryos exhibit early lethality due to defects in both the embryonic mesendoderm and the extraembryonic trophoblast cell lineage. In contrast, zebrafish lacking the predominant Eomes homologue A (Eomesa) do not suffer complete lethality and can be maintained. This suggests fundamental differences in either the molecular function of Eomes orthologues or the molecular configuration of processes in which they participate. To explore these hypotheses we initially analysed the expression of distinct Eomes isoforms in various mouse cell types. Next we compared the functional capabilities of these murine isoforms to zebrafish Eomesa. These experiments provided no evidence for functional divergence. Next we examined the functions of zebrafish Eomesa and other T-box family members expressed in early development, as well as its paralogue Eomesb. Though Eomes is a member of the Tbr1 subfamily we found evidence for functional redundancy with the Tbx6 subfamily member Tbx16, known to be absent from eutherians. However, Tbx16 does not appear to synergise with Eomesa cofactors Mixl1 and Gata5. Finally, we analysed the ability of Eomesa and other T-box factors to induce zebrafish left-right organiser progenitors (known as dorsal forerunner cells) known to be positively regulated by *vgll4l*, a gene we had previously shown to be repressed by Eomesa. Here we demonstrate that Eomesa indirectly upregulates *vgll4l* expression via interlocking feedforward loops, suggesting a role in establishment of left-right asymmetry. Conversely, other T-box factors could not similarly induce left-right organiser progenitors. Overall these findings demonstrate conservation of Eomes molecular function and participation in similar processes, but differential requirements across evolution due to additional co-expressed T-box factors in teleosts, albeit with markedly different molecular capabilities. Our analyses also provide insights into the role of Eomesa in left-right organiser formation in zebrafish.

## Introduction

T-box transcription factors (TFs) are an ancient family of transcriptional regulators with diverse roles in development and disease (Papaioannou, 2014). Eomesodermin (Eomes) belongs to the Tbr1 subfamily of T-box TFs, consisting of similarly sized N- and C-terminal domains (NTD and CTD) flanking a central DNA binding domain known as the T-box. Amongst the species where *Eomes* is best studied are mouse and zebrafish (Probst and Arnold, 2017). Mice have a single copy of *Eomes*, whereas zebrafish owing to the whole genome duplication in the teleost lineage have two paralogous genes, *eomesa* and *eomesb* (Glasauer and Neuhauss, 2014). During mouse embryogenesis *Eomes* plays essential roles in trophectoderm (Russ et al., 2000; Strumpf et al., 2005), in the primitive streak for epithelial-to-mesenchymal transition, mesoderm migration and specification of definitive endoderm and cardiac mesoderm during gastrulation (Arnold et al., 2008a; Costello et al., 2011). Additionally *Eomes* acts in the visceral endoderm to control anterior-posterior axis identity (Nowotschin et al., 2013) and later has key functions in cortical neuron progenitors (Arnold et al., 2008b). It is also expressed in progenitors of the left-right organiser known in mammals as the node, and is required for correct formation of the node suggesting a potential role in establishing left-right asymmetry (Arnold et al., 2008a; Costello et al., 2011). In zebrafish Eomesa also plays multiple roles in mesendoderm formation. It acts in conjunction with Hwa to control expression of Nodal pathway ligands *ndr1/2*, leading to mesendoderm induction (Xing et al., 2022). Eomesa can also induce ectopic endoderm if overexpressed with essential interacting factors (Bjornson et al., 2005), and is sufficient to induce dorsal mesoderm markers and represses ectoderm gene expression in early development (Bruce et al., 2003; Nelson et al., 2014). Furthermore, Eomesa is sufficient to induce progenitors of the left-right organiser, known as dorsal forerunner cells (DFCs) in zebrafish (Bjornson et al., 2005). However, we previously found that Eomesa represses expression of the transcriptional cofactor *vgll4l* (Nelson et al., 2014), a key positive regulator of DFC proliferation, survival and function (Fillatre et al., 2019). Here we further investigate these paradoxical findings.

Mouse *Eomes* and zebrafish *eomesa* display similar expression domains during early development (Russ et al., 2000; Mione et al., 2001; Pearce et al., 2003; Takizawa et al., 2007; Du et al., 2012; Takizawa et al., 2014). However, surprisingly endoderm, cardiac mesoderm and axial patterning proceed normally in *eomesa* loss-of-function mutants (Du et al., 2012). This observation cannot be explained simply by rescue by *eomesb*, which is not co-expressed with *eomesa* in early development, nor is it induced in *eomesa* mutant embryos (Vesterlund et al., 2011; Nelson et al., 2014). The extent to which Eomes functional activities are conserved between zebrafish and mouse remains unknown.

One possibility is that these distinct loss-of-function phenotypes could potentially be due to functional diversification during evolution. The process of alternative splicing (AS) allows a single gene to give rise to multiple isoforms with different functional characteristics. The prevalence of AS has expanded across evolutionary time, allowing increased proteome diversity out of proportion with gene number (Keren et al., 2010). For example, only ∼25% of nematode genes have alternative isoforms compared to >90% in human (Wang et al., 2008; Ramani et al., 2011). AS leading to functional diversification may account for altered functions of Eomes between species. However, it is also possible that differential requirement for Eomes is due to functional redundancy owing to altered complements of T-box factors in different vertebrate evolutionary lineages. The most ancient T-box factor *Brachyury* (otherwise known as *Tbxt*) is present in several non-metazoan lineages, however, the T-box family is considerably expanded in Metazoa, reflecting its developmental importance (Sebe-Pedros et al., 2013). Additionally, the complement of T-box factors has varied across vertebrate evolution, with gain or loss of individual factors in certain lineages. For example, the Tbx6 subfamily member *tbx16* is present in fish, frogs, birds, marsupials and monotremes but lost in placental mammals (Ahn et al., 2012). The T-box domain itself directly binds DNA in a sequence-specific manner. Genome-wide profiling of multiple T-box factors including Eomes, Tbx16, Tbx6 and Brachyury in zebrafish, mice, *Xenopus* and human has revealed they bind most frequently to variants of an eight to nine base pair core consensus of (T)TVRCACHT, interchangeably allowing occupancy of different T-box factors at the same genomic sites e.g. (Morley et al., 2009; Teo et al., 2011; Nelson et al., 2012; Gentsch et al., 2013; Lolas et al., 2014; Nelson et al., 2014; Faial et al., 2015; Tsankov et al., 2015; Windner et al., 2015; Gentsch et al., 2017; Nelson et al., 2017). T-box factors therefore often exhibit redundancy through regulation of the same target genes through the same *cis*-regulatory modules.

We therefore sought to answer three key questions: 1. Are zebrafish and mouse *Eomes* genes functionally equivalent? 2. What is the basis for the observed differences in severity of loss-of-function phenotypes between mouse and zebrafish? and; 3. How can Eomesa promote DFC gene expression while repressing the key DFC regulator *vgll4l*?

Our analyses suggest that the molecular function of *Eomes* is highly conserved throughout vertebrate evolution. Our data also reveal that while alternative splicing of mouse *Eomes* transcript occurs at exon 6, functionally the encoded proteins were virtually indistinguishable. We found that Eomesa and Tbx16 share overlapping functions and capabilities in the presumptive endoderm, suggesting that phenotypic rescue by Tbx16 may explain *eomesa* mutant viability. Finally, we found that Eomesa acts within interlocking feedforward loops to both repress *vgll4l* and activate it indirectly via the essential SOX family transcription factor Sox32. Our results therefore advance our understanding of T-box factor functional conservation during early vertebrate embryogenesis, and regulatory networks controlling left-right organiser progenitor gene expression.

## Materials and methods

### Zebrafish strains

AB and mutant zebrafish were reared as described (Westerfield, 2000). For *eomesa* mutant experiments eggs from homozygous *eomesa^fh105/fh105^* females were *in vitro* fertilized with *eomesa^+/fh105^* sperm yielding a mixture of M*eomesa* and MZ*eomesa* mutant embryos. Since previous studies have revealed no differences in endodermal or mesodermal expression between M*eomesa* and MZ*eomesa* mutant embryos we did not distinguish between them in this study (Du et al., 2012; Xu et al., 2014). All zebrafish studies complied fully with the United Kingdom Animals (Scientific Procedures) Act 1986 as implemented by King’s College London, The University of Warwick, or were in accordance with the policies of the University of Toronto Animal Care Committee.

### Cloning for *in vitro* production of mRNAs and mammalian expression vectors

Full length *tbx16* and *eomesb* open reading frames were cloned with C-terminal myc tags into XhoI and XbaI sites in pCS2+ by PCR from zebrafish cDNA using the following primers: *tbx16-myc* CAT​ACT​CGA​GAT​GCA​GGC​TAT​CAG​AGA​CCT and CGC​GTC​TAG​ACT​ACA​GAT​CCT​CTT​CTG​AGA​TGA​GTT​TTT​GTT​CCC​AGC​ACG​AGT​ATG​AGA​AAA; *eomesb-myc* ATA​TCT​CGA​GAT​GCC​CGG​AGA​AGG​ATC​CAG and GCG​CTC​TAG​ACT​ACA​GAT​CCT​CTT​CTG​AGA​TGA​GTT​TTT​GTT​CGC​TGC​TGG​TGT​AGA​AGG​CGT​A. Full length *gata5* cDNA with a C-terminal HA tag was similarly cloned into pCS2+ EcoRI and XhoI sites using primers CGC​CGA​ATT​CAT​GTA​TTC​GAG​CCT​GGC​TTT and AAT​GCT​CGA​GTC​AAG​CGT​AAT​CTG​GAA​CAT​CGT​ATG​GGT​ACG​CTT​GAG​ACA​GAG​CAC​ACC. *Eomes* cloning into pCS2+ was performed between EcoRI and XhoI sites. pCS2+*eomesaN320K* was produced by PCR mutagenesis of the wild type construct using AAA​CTG​AAG​CTA​ACC​AAC​AAG​AAA​GGA​GCA​AAT​AAC​AAC​AAT and TCC​GAA​AGA​TAT​TTC​TTG​TCT followed by recircularization. *Eomesa* ∆CTD was similarly produced using the following primers TAA​GAA​CTG​CTT​TTC​AAG​ATC​CTT​TAT​CAA​TCC and CGA​ATC​ATA​ATT​GTC​CCT​GAA. The ∆NTD mutation was produced by removing the EcoRI/BstEII fragment from pCS2+*eomesa* and replacing with the EcoRI/BstEII fragment produced by PCR from pCS2+*eomesa* with primer pair GCC​CTC​GAA​TTC​ACA​GTT​AAG​AAT​GGC​GCG​GGC​GC and CCC​GCA​GGT​CAC​CCA​CTT​TCC​GCC​CTG​AAA​TCT​CCA.

### mRNA, morpholinos and microinjections

All capped mRNA were synthesized from plasmids encoding proteins of interest in pCS2+ NotI linearization followed by SP6 transcription as described (Bruce et al., 2003), with the exception *tbxta* which was produced from pSP64T as described (Marcellini et al., 2003). mRNA quantities for T-box factors were scaled in order to inject equimolar amounts of each mRNA per embryo. One-cell stage embryos were injected with the following quantities: *eomesa* – 400pg; *Eomes∆VR* – 410 pg; *EomesFL* – 420 pg; *eomesa∆NTD* – 308 pg; *eomesa∆CTD* – 285 pg; *eomesaN320K* – 400 pg; *eomesb-myc* – 286 pg, *tbx16-myc* – 217 pg; *tbxta* – 223 pg; *gata5-HA* – 140 pg; *mixl1* – 200 pg. For Tbx16 knockdown one-cell stage embryos were injected with, 0.5 pmol of a previously characterized *tbx16* morpholino (GeneTools) (Bisgrove et al., 2005).

### *In vitro* protein production

Unlabelled *in vitro* translated protein was produced using rabbit reticulocyte lysates according to manufacturer’s protocol (Promega).

### Northern blot

Total RNA was extracted from specified cell types using Rneasy Mini Kits (QIAGEN), and polyA selected using Oligotex mRNA Mini Kits (QIAGEN). 500ng polyA + RNA per lane was size fractionated on a 1.5% agarose/MOPS gel, transferred onto Hybond N membranes (GE Healthcare), and probed with ^32^P-random-primed 1 kb XmaI-EcoRV cDNA fragment corresponding to the exon 1–4T-box region.

### Western blot

Cell lysates were prepared using radioimmunoprecipitation assay (RIPA) buffer, subjected to SDS–polyacrylamide gel electrophoresis and transferred onto polyvinylidene difluoride membranes. Membranes were blocked with 5% milk powder in Tris-buffered saline with Tween 20, incubated in primary antibodies overnight including rabbit anti-Eomes CTD (Abcam, ab23345, 1:2,000), rabbit anti-Eomes NTD (Santa Cruz, sc-98555, 1:1,000) and rat anti-Eomes (eBioscience, 14–4,876, 1:1,000). Secondary antibodies were donkey anti-rabbit horseradish peroxidase (GE Healthcare NA934, 1:2,000) and goat anti-rat horseradish peroxidase (GE Healthcare NA935, 1:2,000). Blots were developed by chemiluminescence using Amersham ECL Prime Detection Reagent (GE Healthcare).

### Embryonic stem cell differentiation

Wild type (+), *Eomes* ^*null/null*^ (null), feeder-depleted ESCs were cultured in DMEM (ThermoFisher) with 15% FCS, 1% non-essential amino acids, 0.1 mM β-mercaptoethanol and 1,000 U/ml recombinant leukaemia inhibitory factor (Millipore). For differentiation ESCs were resuspended at 1×10^4^ cells/ml in DMEM (ThermoFisher) with 15% FCS, 1% non-essential amino acids, 0.1 mM β-mercaptoethanol in hanging drops (10 μL) plated on the inside lids of bacteriological dishes. After 48 h embryoid bodies were transferred in 10 ml medium to 10 cm bacteriological dishes and RNA extracted at the appropriate timepoints.

### P19Cl6 cell culture and differentiation

P19Cl6 embryonal carcinoma cells were cultured in α-MEM (ThermoFisher) supplemented with 10% FCS. To induce differentiation, cells were seeded at 3.7×10^5^ cells/6 cm dish in media containing 1% DMSO (Sigma) and RNA harvested after 72 h.

### Cytotoxic T-cell lymphocyte, neomycin- and hygromycin B-resistant STO fibroblasts (SNH) and HeLa cell culture

CTLL cells derived from the ATCC TIB-214 line were maintained at 10^4^–10^5^ cells per ml in complete T cell medium supplemented with IL-2. SNH fibroblasts and HeLa cells were maintained on 0.1% gelatin coated dishes in DMEM supplemented with 10% bovine calf serum (Hyclone).

### Reverse transcription–polymerase chain reaction

Cytoplasmic RNA was produced as previously described (Eggermont and Proudfoot, 1993). Total RNA was produced using Rneasy Mini Kits according to manufacturers protocol (QIAGEN). RT-PCR was performed using OneStep RT-PCR Kit (QIAGEN) using the following primers: Total *Eomes*–TGTTTTCGTGGAAGTGGTTCTGGC and AGG​TCT​GAG​TCT​TGG​AAG​GTT​CAT​TC; *Eomes* exon 4-6 to distinguish FL and ∆VR isoforms ATC​GTG​GAA​GTG​ACA​GAG​GAC​G and CGG​GAA​GAA​GTT​TTG​AAC​GCC; Gapdh–TGCACCACCAACTGCTTAGC and GGC​ATG​GAC​TGT​GGT​CAT​GAG; *Eomes* start codon to ∆CTD 3′ UTR–ATATCTCGAGATGCAGTTGGGAGAGCAGCTC and TGG​GCT​CGA​AGA​TGA​AAC​TC; *HBB* exon 2 to *Eomes* exon 6 – GCA​CGT​GGA​TCC​TGA​GAA​CT and CGG​GAA​GAA​GTT​TTG​AAC​GCC. For nested PCR to test exon 5-6 splicing association with the long *Eomes* 3’ UTR the initial primer pair used was ATC​GTG​GAA​GTG​ACA​GAG​GAC​G and CAA​GTA​CGG​AGG​CAG​CTG​AG.

### Whole-mount embryo staining

Whole-mount *in situ* hybridization (WISH) of zebrafish embryos were performed as described (Jowett and Lettice, 1994). Anti-sense riboprobes for *noto* (Talbot et al., 1995), *chrd* (Miller-Bertoglio et al., 1997), *vgll4l* (Nelson et al., 2014), *zic3* (Grinblat and Sive, 2001)*, mixl1* (Alexander et al., 1999) and *sox32* (Dickmeis et al., 2001) were produced as described. Blinding and randomisation was performed prior to categorical scoring of WISH embryos to prevent bias.

### Cloning and mutagenesis to test eomes exon 6 splice sequences

Clones to test the splicing efficiency at *Eomes* exon 6 were generated by cloning PCR products using primers ACG​GCA​ATT​GGC​CTC​GAA​CAT​TCT​TGC​TTC and CCA​GCC​ATC​ACT​TTG​GTC​AAA​GGT​GGA​AGG​CAA​AAG into MfeI-BstXI sites of the human *HBB* gene (GenBank accession no. U01317) within a previously described TAT-inducible expression vector (Ashe et al.,1997). Mutation of splicing sequences for the ∆VR and FL *Eomes* isoforms were introduced by PCR using primers: ∆VR - CAT​GTA​CAC​GGC​TTC​AGA​AAA​CGA​CAG​GTT​AAC​GCC​AAG​TCC​GAC​GGA​TTC​CCC​TCG​ATC​CCA​TCA​GAT​TGT​CCC​TGG and CTA​CAA​TAT​AAA​GAG​AGA​CAC​TTA​AAA​ATA​AAA​AAC​AAC​CCT​CAC​GTT​GTC​CCC​AAA​CAA​GCT​GCC​TCC​CAG​AAG​C; FL–CATGTACACGGCTTCAGAAAATGACAGGTTAACTCCA TCTCCCACGGATTCCCC and GGA​CAT​TAT​ATA​CAC​CGC​CTC​TTA​TAT​TTT​TAC​ACC​AAC​CCT​CAC​GTT​GTC​CCC​AAA​CAA​GCT​GCC​TCC​CAG​AAG​C followed by recircularization of the resulting PCR products. Deletion of the VR was similarly performed using primers ATC​CCA​TCA​GAT​TGT​CCC​TGG​A and CTA​CAA​TAT​AAA​GAG​AGA​CAC​TTA​AAA​ATA​AAA​AAC​AAC​CCT​CAC​GTT​GTC​CCC​AAA​CAA​GC T​GCC​TCC​CAG​AAG​C. *HBB* plasmids were co-transfected with a plasmid expressing the HIV transactivator protein TAT (Adams et al., 1988), into HeLa cells using Lipotectamine 2000 according to manufacturers protocol (ThermoFisher).

### Conservation analysis

The Tbr1 subfamily Gene Tree was generated by Ensembl (Yates et al., 2016). *Eomes* conservation measurements (*phyloP*) across 60 vertebrate species were visualized in UCSC Genome Browser (http://genome.ucsc.edu/) (Karolchik et al., 2004; Speir et al., 2016). Sequence logos of the Eomes variant region in placental mammals, other tetrapods and teleosts were based on alignment of the same 60 vertebrate species and visualized using WebLogo (Crooks et al., 2004).

Full-length protein alignments were performed using Clustal Omega (Goujon et al., 2010; Sievers et al., 2011; McWilliam et al., 2013) and visualized using JalView (Waterhouse et al., 2009). BLOSUM62 average distance gene tree was also produced using Jalview.

### Single-cell ribonucleic acid sequencing analysis

Single-cell (sc) RNA sequencing count data of zebrafish embryonic cells from Wagner *et al* (2018) was downloaded from GEO (Barrett et al., 2013; Wagner et al., 2018). Raw UMI-filtered count data in CSV format from 6 h post fertilisation (h.p.f.) embryos (GSM3067190) was imported in to R v3.6.2 and analysed using Seurat v3.0.2 (Stuart et al., 2019). Cells with less than 200 features, and features detected in <3 cells were discarded. The remaining count data were then normalised via SCTransform v0.2.1 (Hafemeister and Satija, 2019) with mitochondrial genes passed as a regression variable. Genes were clustered using UMAP utilising the R package uwot v0.1.5 (Melville et al., 2020), with the following parameters: dims = 1:30, n.neighbors = 5, min.dist = 0.001. To assign cell identities to clusters FindAllMarkers was called in Seurat using default parameters. For consistency with the original source publication of the 6 h.p.f. scRNA-seq data we cross-referenced the marker genes for each cluster in the present study with the clusters defined by Wagner *et al.* (2018). Significant positive markers in each of the 14 clusters defined by our analyses in Seurat were overlapped with the top 20 markers for each identity defined by Wagner *et al.* (2018). Cell identities were then assigned based on maximum concordance with markers defined by Wagner *et al.* (2018).

## Results

### Eomes isoforms, conservation and expression

**FIGURE 1:**
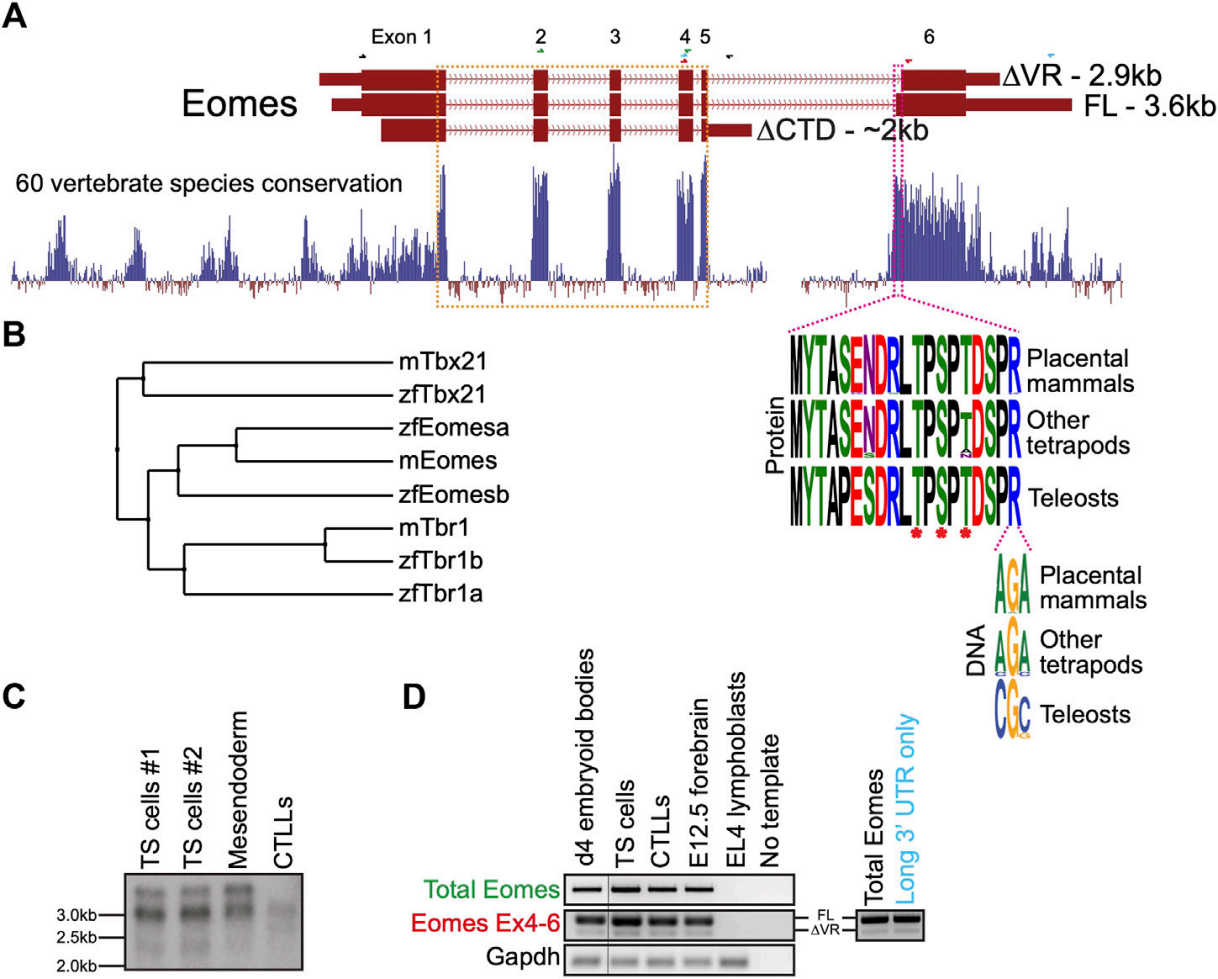
Mouse *Eomes* has multiple isoforms, including a mammalian-specific alternative splicing event. **(A)** Gene model with conservation track and sequence logos for variant region. All transcripts are Ensembl version 107 annotations - ∆VR transcript is ENSMUST00000111763; FL transcript is ENSMUST00000035020; ∆CTD transcript is ENSMUST00000150633. Annotated transcript sizes are indicated, as well as amino acid conservation of the VR between placental mammals, other tetrapods and teleosts, and the variation within the terminal VR arginine codon. The VR is defined by the amino acids present in ENSMUSP00000035020 (encoded by ENSMUST00000035020) that are absent from ENSMUSP00000107393 (encoded by ENSMUST00000111763). The T-box is outlined in orange and the VR in pink. Asterisks indicate known phosphorylated amino acid residues. RT-PCR primer pairs are indicated as half arrows and colour-coded as follows: black–to establish connectivity between the annotated start codon and ∆CTD isoform 3′ UTR; green–to assess total Eomes through amplification of exon 2–4; blue–to amplify Eomes cDNA between exon 4 and the distal 3′ UTR; red–to assess alternate splicing at exon 6. **(B)** BLOSUM62 average distance evolutionary tree of the Tbr1 subfamily showing relationships between mouse and zebrafish genes. **(C)** Northern blot showing Eomes transcripts in different cell types using a probe against the T-box. Data for two independent trophoblast stem (TS) cell lines are shown. Mesendoderm is P19Cl6 cells after 4 days of DMSO induced differentiation. CTLLs are IL-2-dependent T-cell lymphocytes derived from ATCC TIB-214. **(D)** RT-PCR showing relative levels of FL and ∆VR isoforms (left), and nested PCR showing FL/∆VR ratio for long 3′ UTR transcripts (right). Day 4 differentiated embryoid bodies contain cells mimicking embryonic endoderm. CTLLs are IL-2-dependent T-cell lymphocytes derived from ATCC TIB-214. EL4 cells are a negative control for *Eomes* expression. *Gapdh* is a loading control. Locations of primer pairs used for RT-PCR are shown in panel A and the text colour-coded accordingly. Nested PCR to analyse exon 6 splicing in transcripts containing the long 3′UTR was performed using the blue primer pair in panel A, followed by the red primer pair.

In mouse the three annotated *Eomes* transcripts give rise to three structurally distinct isoforms including the full length (FL) product, a splice variant having an alternative splice acceptor site within exon 6 leading to loss of a 19 amino acid variant region (∆VR), and a transcript with an alternative mRNA 3’ cleavage site leading to loss of exon 6 and its encoded CTD (∆CTD) (Figure 1A). The highly conserved VR sequence is known to be phosphorylated at three amino acid residues in mouse spleen and kidney (Figure 1A) (Huttlin et al., 2010). The internal exon 6 splicing event emerged in the tetrapod lineage through a synonymous single nucleotide change in an arginine codon (CG>AG) introducing a splice acceptor sequence.

Because the ∆CTD transcript annotation has an incomplete 5′ end, it remains unclear whether it encodes the entire NTD. However, our RT-PCR analysis using primers located in the ∆CTD 3’ UTR and at the FL/∆VR start codon suggests that exon 1 coding information is intact (not shown). The CTD encoded by exon 6 has been shown to function in transcriptional activation (Picozzi et al., 2009), suggesting that the ∆CTD isoform is likely to be functionally compromised in comparison to FL and ∆VR isoforms. Consistent with this, the CTD is more highly conserved than the NTD (Figure 1A). Functional differences between FL and ∆VR isoforms, however, are yet to be examined. Both *eomesa* transcripts identified in zebrafish encode the same protein (Bruce et al., 2003). Relatively little is known about the single annotated zebrafish *eomesb* transcript, which appears to be more divergent from the ancestral gene (Figure 1B).

Murine *Eomes* is expressed in numerous cell types including trophoblast stem cells (TSCs), mesendoderm, and T lymphocytes. To identify *Eomes* transcripts we initially performed Northern blot analysis (Figure 1C). Transcript sizes corresponding to all three annotated isoforms were detectable but the ∆CTD transcript was underrepresented. The FL and ∆VR annotations display different 3′ UTR lengths. To test whether the two distinct upper bands detected by Northern blot correspond to alternative exon 6 splicing events or merely different UTR lengths we next performed nested PCR (Figure 1D). We found that the long 3′ UTR is associated with both the FL and ∆VR coding isoforms. Strikingly, the ratio of FL/∆VR is similar for both total Eomes and the long 3’ UTR transcripts. The abundance of the different coding transcripts therefore appears to be independent of UTR length. Further analysis through cloning *Eomes* intron5/exon6 to replace intron2/exon3 of the human *HBB* gene in an expression construct followed by transfection into HeLa cells revealed that the ratio of FL/∆VR splicing is consistent with the wild type *Eomes* gene, suggesting that the low levels of the ∆VR isoform are due to weaker splicing consensus sequences, thus favouring the FL isoform (Supplementary Figure S1). However, analysis of Eomes proteins by Western blot indicates that various N-terminal truncations occur which cannot be accounted for by the annotated coding transcripts (Supplementary Figure S2). We conclude that FL is clearly the most abundant of the three annotated coding isoforms. Importantly, this predominant isoform expressed by mouse cells corresponds to the single *eomesa* isoform in zebrafish.

### Both mouse full length and ∆VR isoforms are functionally equivalent to zebrafish *eomesa* in early development

Zebrafish *eomesa* loss of function causes less severe phenotypes compared with the dramatic defects observed in *Eomes* mutant mouse embryos. To further explore mouse and zebrafish Eomes functional capabilities we overexpressed either mouse *Eomes* FL or ∆VR mRNAs in zebrafish embryos for comparison with those overexpressing zebrafish *eomesa.*

**FIGURE 2:**
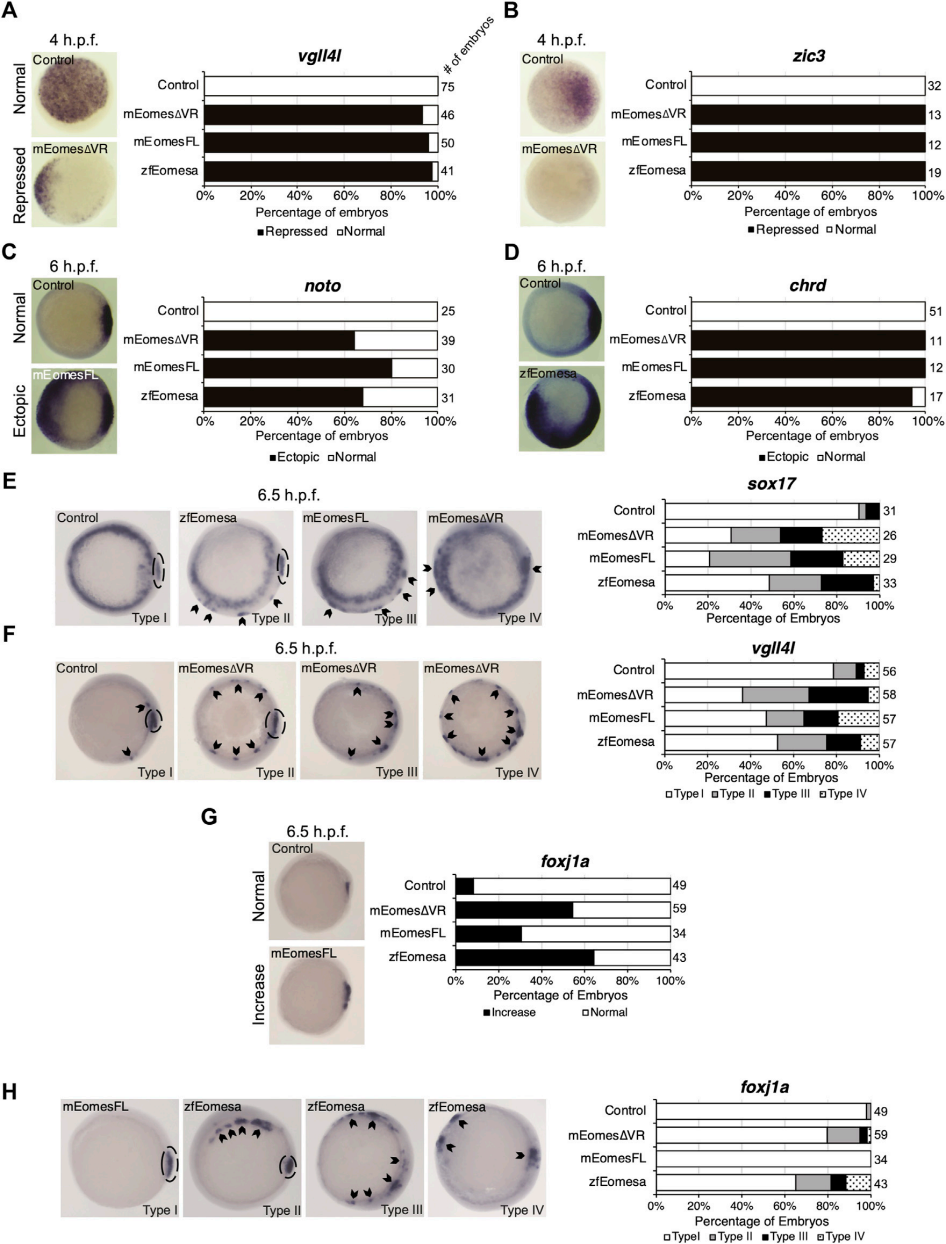
Both FL and ∆VR isoforms of mouse Eomes are functionally equivalent to zebrafish Eomesa in the early embryo. WISH analysis of ectoderm markers *vgll4l* and *zic3* in mid/late blastulas (4 h.p.f.) embryos **(A,B)**, organiser markers *noto* and *chrd* **(C,D)** in early gastrulas (6 h.p.f.), or DFC markers *sox17*, *vgll4l* and *foxj1a* **(E–H)** in early/mid gastrulas (6.5 h.p.f.). Embryos have been injected at the 1 cell stage to overexpress either mouse EomesFL, Eomes∆VR or zebrafish Eomesa. *N* = 2. Total numbers of embryos scored per condition are indicated. Representative images of expression patterns per gene per category are shown. **(A–D)** Animal views; dorsal to the right. **(E)** Animal views; dorsal to the right. **(F–H)** Vegetal views; dorsal to the right. Panel G indicates the percentage of embryos with greater intensity of dorsal *foxj1a* WISH staining, while panel H indicates percentages of embryos with ectopic *foxj1a* staining. Type I–wild type expression; Type II–ectopic dorsolateral expression with clear primary dorsal DFC cluster; Type III–dorsolateral expression with no defined primary cluster; Type IV–ectopic expression in the ventral margin. Dotted ovals indicate primary DFC clusters. Arrowheads indicate ectopic DFC marker expression.

Zebrafish Eomesa represses ectoderm genes such as *vgll4l* and *zic3* during blastula stages and activates mesendoderm genes including organizer markers *noto* and *chrd* at the onset of gastrulation (Bruce et al., 2003; Nelson et al., 2014). Injecting equimolar quantities of zebrafish *eomesa* mRNA, or mouse FL or ∆VR Eomes isoforms at the one-cell stage, we found that each was equally able to repress *vgll4l* and *zic3* in mid/late blastulas (4 h post-fertilisation - h.p.f.) and induce *noto* and *chrd* in early gastrulas (6 h.p.f.; Figures 2A–D).

Zebrafish Eomesa is suggested to induce DFCs, based on observation of ectopic *sox17* expression in cells of the outer margin in early gastrulas (7 h.p.f.) on *eomesa* overexpression (Bjornson et al., 2005). We sought to determine whether these ectopic *sox17* + cells express a broader range of DFC markers (*sox17*, *vgll4l* and *foxj1a*), and also whether they can be similarly induced by mouse Eomes. Notably, though Eomesa represses expression of *vgll4l* during blastula stages, during gastrulation *vgll4l* is expressed in DFCs, and has recently been identified as a key regulator of DFC proliferation, survival and function (Fillatre et al., 2019). Repression of *vgll4l* at later stages in DFCs would consequently be inconsistent with promoting DFC formation.

We found that *eomesa*, and *Eomes* FL or ∆VR isoforms were all similarly able to upregulate both *sox17* and *vgll4l* in the outer margin of gastrulas. We further note that there was a diversity of phenotypes beyond wild type expression (type I) wherein ectopic dorsolateral expression was markedly observed in individual cells/small clusters outside the primary dorsal DFC cluster (type II), where there was dorsolateral expression with no defined primary cluster (type III), and where ectopic expression was also observed in the ventral margin (type IV) (Figures 2E,F). Conversely, for *foxj1a* we note that while *eomesa*, *Eomes* FL and ∆VR can induce a greater intensity of dorsal staining, and expansion of the dorsal DFC cluster, ectopic expression in the ventrolateral margin is rare compared to other DFC markers (Figures 2G,H).

We conclude that both FL and ∆VR mouse Eomes isoforms are functionally highly similar to zebrafish Eomesa in these contexts. Moreover, Eomesa regulation of *vgll4l* appears to be context-specific, repressing its expression during blastula stages while inducing its expression in DFCs during gastrulation. We further conclude that additional factors likely to be predominantly dorsally localised are required for robust upregulation of *foxj1a* compared to *sox17* and *vgll4l*.

### Eomesa and non-mammalian T-box factor Tbx16 redundantly regulate *mixl1* expression at the initiation of zebrafish endoderm formation

**FIGURE 3:**
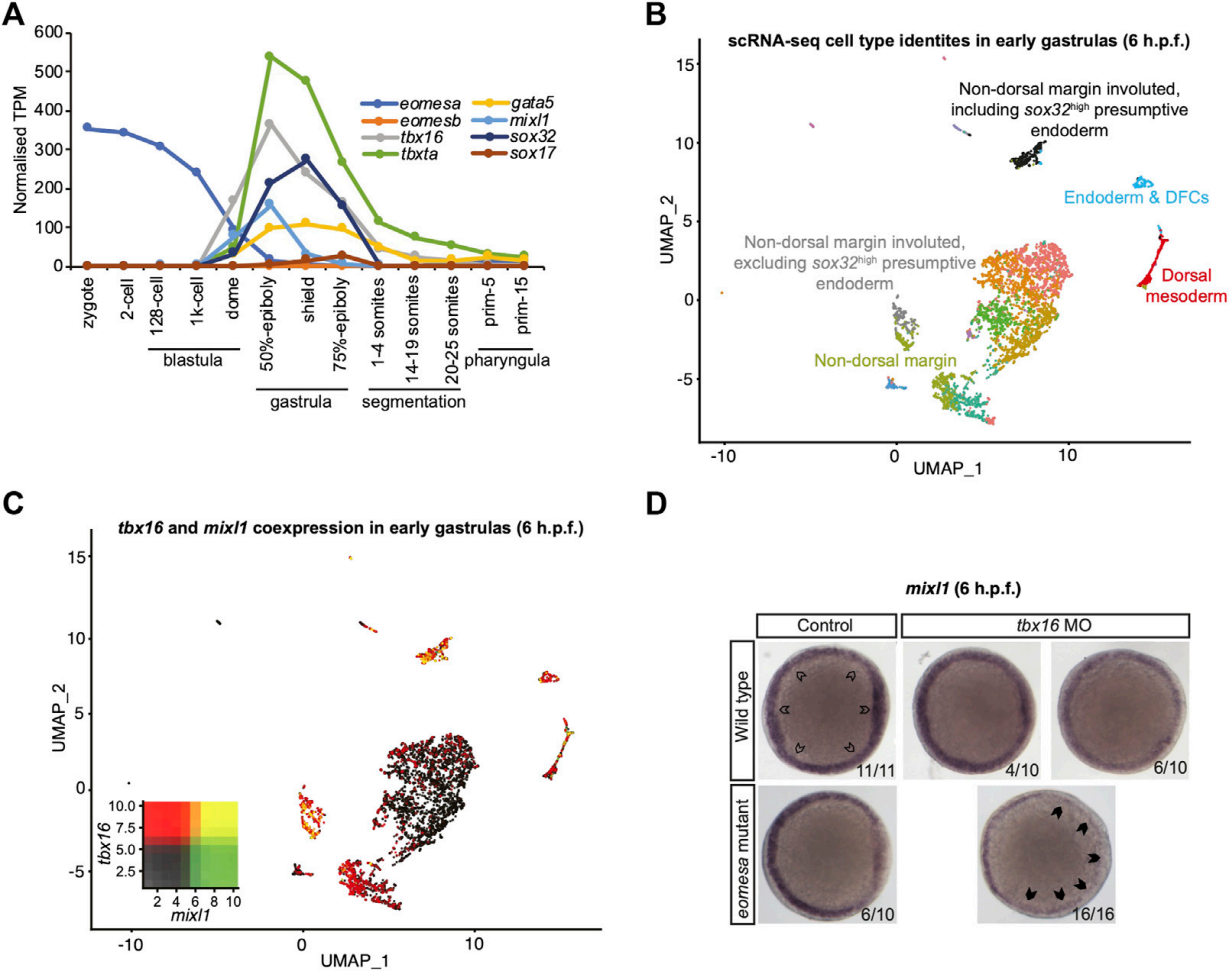
Eomesa and Tbx16 are redundantly required for *mixl1* expression in the presumptive endoderm. **(A)** Timing of expression of T-box factors (*eomesa*, *eomesb*, *tbxta*, *tbx16*) under study and presumptive endoderm (*mixl1*, *gata5*, *sox32*) and endoderm markers (*gata5*, *sox32*, *sox17*) indicated by bulk RNA-seq data from (White et al., 2017). Gene expression is shown as transcripts per million (TPM). Stages are as defined by (Kimmel et al., 1995). **(B)** UMAP clustering analysis of single-cell RNA-seq data for early gastrulas (6 h.p.f.) zebrafish embryos (Wagner et al., 2018) indicating colour-coded cell type identities. Cell types relevant to the present study are labelled. The identities of all cell types are indicated in Supplementary Figure S3. **(C)** UMAP clustering analysis of single-cell RNA-seq data for early gastrulas (6 h.p.f.) zebrafish embryos indicating co-expression of tbx16 and mixl1 (Wagner et al., 2018). Heatmap insets indicate overall expression levels per gene and co-expression. Overlapping expression is shown in yellow. **(D)** WISH analysis of *mixl1* in early gastrulas (5.7–6 h.p.f.) zebrafish embryos in wild type or eomesa mutant embryos (see Methods for information on genotype) with and without Tbx16 morpholino knockdown. Total numbers of embryos and fractions as categorised are indicated. Animal views; dorsal to the right. Open arrowheads indicate normal mixl1 expression at the blastoderm margin. Closed arrowheads indicate profound loss of *mixl1* expression on *tbx16* knockdown in *eomesa* mutants.

Since results above strongly suggest mouse Eomes is functionally similar to zebrafish Eomesa, do *eomesa* mutants have comparatively mild defects due to functional redundancy with other T-box factors? *Eomesb* is not appreciably expressed during early zebrafish development (Figure 3A), and is not upregulated in *eomesa* mutants (Nelson et al., 2014) thus it seems unlikely that it compensates for loss of Eomesa. We recently identified a key role for the non-placental mammal T-box factor, Tbx16 in endoderm formation, with Brachyury homologue Tbxta having a more minor role (Nelson et al., 2017). Both of these factors show zygotic upregulation concomitant with declining *eomesa* mRNA levels and prior to expression of key markers of presumptive endoderm (e.g., *gata5* and *mixl1*), and endoderm (e.g., *gata5*, *sox32* and *sox17*; Figure 3A), thus may compensate for the early loss of expression of such markers in MZ*eomesa* mutants (Du et al., 2012).

Moreover, single-cell RNA-seq data (Wagner et al., 2018) demonstrate that *tbx16* is robustly co-expressed with the critical endodermal regulator *mixl1* in the presumptive endoderm at early gastrulation stages (6 h.p.f.), suggesting the potential for Tbx16 to upregulate *mixl1* expression to initiate endoderm specification, as our previous published analyses suggest (Nelson et al., 2017) (Figures 3B,C). Additional specific detail on cluster identities in Figures 3B,C is provided in Supplementary Figure S3.

To test whether Tbx16 functions redundantly with Eomesa during endoderm formation next we performed antisense morpholino knockdown of Tbx16 in wild type and *eomesa* mutant embryos. We found that while *mixl1* expression is reduced on loss of Eomesa or Tbx16 alone, loss of both TFs leads to more striking loss of *mixl1* (Figure 3D). Eomesa and Tbx16 therefore collaboratively activate *mixl1* expression, strongly suggesting that Tbx16 relieves the requirement for Eomesa in zebrafish endoderm formation.

### *Tbx16* and *eomesa* overexpression do not equivalently induce endoderm fate in concert with *mixl1* and *gata5*

**FIGURE 4:**
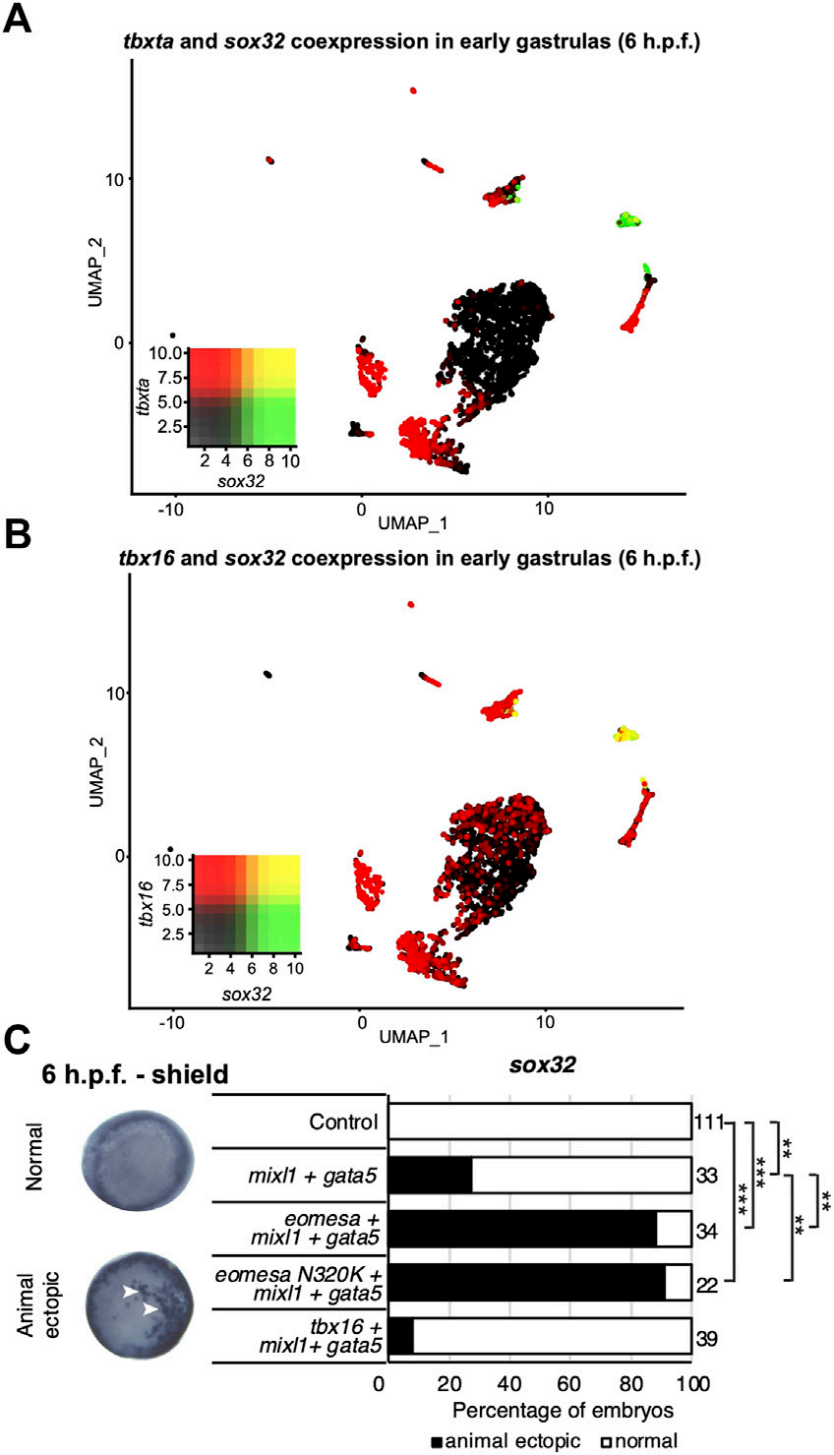
Tbx16 is substantially co-expressed with *sox32* but cannot induce it in combination with *mixl1* and *gata5*. **(A,B)** UMAP clustering plots of whole embryo single-cell RNA-seq data in early gastrulas (6 h.p.f.) indicating co-expression of *sox32* with *tbxta* and *tbx16*. Heatmap insets indicate overall expression levels per gene and co-expression. Overlapping expression is shown in yellow. **(C)** WISH analysis of the ability of *eomesa*, *eomesa*N320K and *tbx16* in combination with *gata5* and *mixl* to induce *sox32* expression at the animal pole of early gastrulas (6 h.p.f.). *N* = 2. Total numbers of embryos scored per condition are indicated. Representative images of expression patterns per gene per category are shown. Animal views; dorsal to the right. Arrowheads indicate ectopic expression. Fisher’s Exact two-tailed probability test *p* values: **P ≤ 5 × 10^−6^; ***P ≤ 5 × 10^−12^.

Eomesa and Mixl1 bind upstream of endoderm master regulator *sox32* to positively regulate its expression prior to endoderm specification (Nelson et al., 2014; Nelson et al., 2017). Eomesa, Mixl1 and Gata5 can physically interact and their combined overexpression has been shown to induce ectopic endoderm gene expression in late blastulas and early gastrulas (Bjornson et al., 2005). Combined expression of Tbxta with Mixl1 and Gata5, however, is insufficient to induce *sox32* expression (Bjornson et al., 2005). This is consistent with limited co-expression between *tbxta* and *sox32* around the time of endoderm specification (Figure 4A). However, *tbx16* and *sox32* are substantially co-expressed in the endoderm at the onset of gastrulation, (Figures 3B, 4B). Tbx16 is also critical for endoderm progenitor induction (Nelson et al., 2017). We therefore extended our study to test whether Tbx16 can induce ectopic endoderm marker expression in cells at the animal pole (i.e. in cells where Tbx16, Mixl1 and Gata5 are absent in wild type embryos) through co-overexpression with Mixl1 and Gata5, as Eomesa can.

As expected, combined overexpression of *eomesa*, *mixl1* and *gata5* induces ectopic *sox32* expression at the animal pole (Figure 4C). However, *tbx16* did not synergise with *mixl1* and *gata5* to upregulate *sox32* in the animal pole. This may suggest that Tbx16 and Eomesa are not equally capable of forming a complex with Mixl1 and Gata5 to induce endoderm and/or DFC fate, or alternatively that there are other key components of the complex which are capable of interaction with Eomesa/Mixl1/Gata5, but not Tbx16/Mixl1/Gata5. We conclude that Eomesa and Tbx16 perform similar functions in overlapping processes in the developing zebrafish embryo, but appear to do so via distinct molecular mechanisms.

### T-box factors co-expressed with *eomesa* do not share its potent abilities to upregulate dorsal marker genes

Tbx16 and Eomesa lack significant sequence homology, especially outside the T-box domain (Supplementary Figure S4). However, *Xenopus* Eomes and its Tbx16 orthologue VegT have been suggested to display similar specificity in part due to a single shared asparagine residue within the T-box, rendering them functionally distinct from the Tbxta orthologue Xbra, which has a lysine in the equivalent position (Conlon et al., 2001) (Supplementary Figure S4). We therefore sought to address the following questions: 1) Are Tbx16, Tbxta and/or Eomesb capable of inducing Eomesa target genes in early gastrulas; 2) Is the T-box asparagine residue critical for Eomesa function; 3) Are key Eomesa functions dependent on the highly conserved CTD or relatively poorly conserved NTD.

**FIGURE 5:**
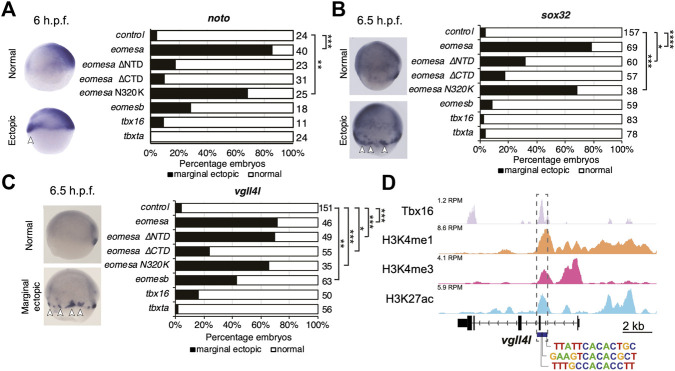
Eomesa is a more potent inducer of endoderm, organiser and dorsal forerunner cell markers than other T-box factors. **(A–C)** WISH analysis of dorsal mesoderm marker *noto* **(A)** and dorsal forerunner cell markers *sox32* **(B)** and *vgll4l* **(C)** on overexpression of various wild type and mutant T-box factors. mRNAs injected at the 1 cell stage; WISH performed at stages as indicated. N = 2. Total numbers of embryos scored per condition are indicated. Representative images of expression patterns per gene per category are shown. Lateral views; dorsal to the right. Arrowheads indicate ectopic expression. Fisher’s Exact two-tailed probability test *p* values: *P ≤ 5 × 10^−4^; **P ≤ 5 × 10^−6^; ***P ≤ 5 × 10^−12^; ****P ≤ 5 × 10^−30^. **(D)** ChIP-seq data in mid/late gastrulas (8–8.5 h.p.f.) indicating Tbx16 binding within the *vgll4l* promoter (Bogdanovic et al., 2012; Nelson et al., 2017). Scale is reads per million reads (RPM). Putative T-box binding sites identified using JASPAR are indicated (Fornes et al., 2020).

We injected equimolar quantities of mRNA corresponding to each wild type T-box factor, or Eomesa ∆NTD, ∆CTD or N320K mutants and assessed the effect on dorsal mesoderm marker *noto* and DFC/endoderm marker *sox32*, and DFC marker *vgll4l*. Deletion of the NTD or CTD ablated Eomesa ability to induce *noto* expression, while N320K mutation had no significant effect (Figure 5A). All other T-box factors failed to produce a consistent or compelling induction of ectopic *noto* (Figure 5A). Consistent with previous results, Eomesa overexpression led to ectopic *sox32* expression in the outer marginal cells indicative of increased numbers of DFC-like cells but not endoderm (Bjornson et al., 2005) (Figure 5B). NTD and especially CTD deletions markedly reduced ectopic *sox32* induction, however, N320K mutation had little effect (Figure 5B). We further note that the N320K mutation had no discernible effect on the ability of Eomesa to synergise with Mixl1 and Gata5 to induce *sox32* expression at the animal pole, suggesting that this mutation within the T-box does not interfere with the known T-box interactions with Mixl1 and Gata5 (Bjornson et al., 2005) (Figure 4C). Overexpression of *tbx16*, *tbxta* and *eomesb* did not lead to ectopic *sox32* induction highlighting functional distinctions with *eomesa* (Figure 5B). *Vgll4l* expression showed similar induction to *sox32* at the margin by both wild type Eomesa and ∆NTD and N320K Eomesa (Figure 5C). We have previously shown by ChIP-seq that at sphere stage Eomesa binds in the first intron of *vgll4l* (Nelson et al., 2014). Analysis of Tbx16 ChIP-seq data in mid/late gastrulas (8–8.5 h.p.f.) (Nelson et al., 2017) also shows Tbx16 binding at close matches to the known T-box consensus sequence within *vgll4l* intron 1, suggesting Tbx16 does have the potential to directly participate in regulation of *vgll4l* during gastrulation (Figure 5D). However, *tbx16* overexpression suggests that it is not individually sufficient to strongly drive ectopic *vgll4l* expression (Figure 5C).

We next tested whether Tbx16 and Tbxta can act combinatorially to induce dorsal marker gene expression. We found that on Tbx16/Tbxta combinatorial overexpression DFC markers *vgll4l* and *sox17* were expressed over a broader region of the dorsolateral margin but showed concomitant reduction in the primary DFC cluster (Supplementary Figure S5A). Conversely, *foxj1a* expression remained localised to the dorsal margin, again suggesting that *foxj1a* expression is somewhat dependent on additional dorsally localised regulators (Supplementary Figure S5A). Furthermore, while *tbx16* and *tbxta* are coexpressed with *noto* in early gastrulas (Supplementary Figures S5B,C), their combinatorial overexpression does not lead to expanded *noto* expression (Supplementary Figure S5D). We therefore conclude that combined activities of Tbx16 and Tbxta are not sufficient to induce dorsal fates, unlike Eomesa.

Overall this suggests that Eomesb, Tbx16 and Tbxta do not individually have similar abilities to Eomesa in inducing dorsal mesoderm and DFC gene expression, that the previously reported N/K amino acid difference between Eomesa/Tbx16 and Tbxta does not appreciably influence specificity and function in this context, and that deletion of the relatively poorly conserved Eomesa NTD does not result in complete loss of function.

### Eomesa regulates *vgll4l* expression and dorsal forerunner cell formation through interlocking feedforward loops via *sox32*

**FIGURE 6:**
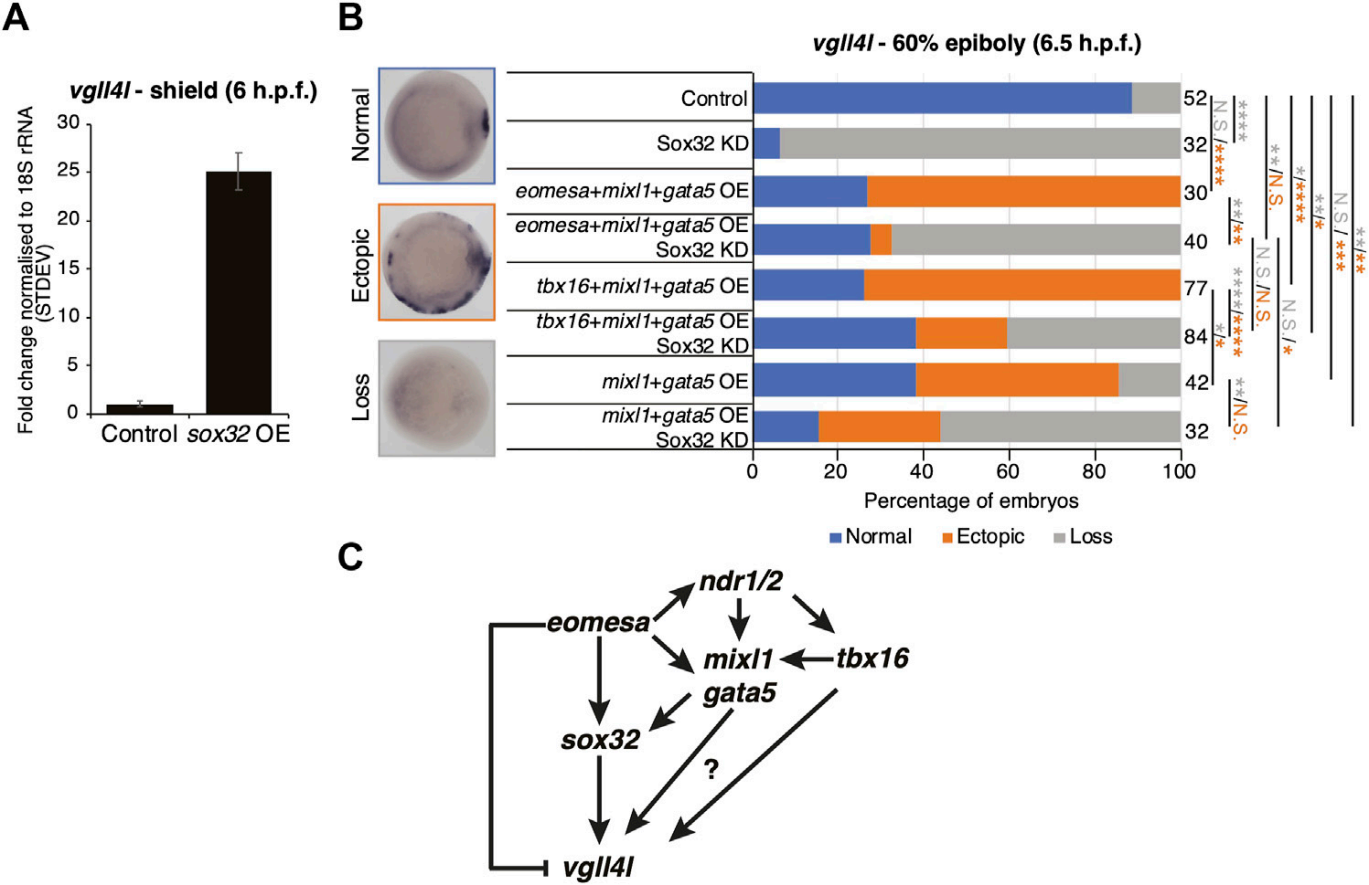
Eomesa activation of *vgll4l* is through feedforward loops via *sox32* and its upstream activators. **(A)** qRT-PCR analysis of *vgll4l* expression in early gastrulas (6 h.p.f.) in control embryos and those injected with *sox32* mRNA at the 1 cell stage. Expression is represented as fold change relative to control normalised to 18S rRNA. **(B)** WISH analysis of the ability of *vgll4l* expression in early gastrulas (6 h.p.f.) in embryos injected with mRNAs at the one-cell stage as indicated, with and without Sox32 morpholino knockdown. *N* = 2. Total numbers of embryos scored per condition are indicated. Representative images of expression patterns per gene per category are shown. Animal views; dorsal to the right. Fisher’s Exact two-tailed probability test *p* values: *P ≤ 5 × 10^−2^; **P ≤ 5 × 10^−4^; ***P ≤ 5 × 10^−8^; ****P ≤ 5 × 10^−12;^ N.S. = not significant. Orange asterisks indicate significant differences in fractions of embryos exhibiting ectopic expression vs. other categories. Grey asterisks indicate significant differences in fractions of embryos exhibiting loss of expression vs. other categories. **(C)** Model for Eomesa regulation of vgll4l expression in dorsal forerunner cells indicating a type 3 incoherent feedforward loop on the left as Eomesa represses vgll4l directly while activating via Sox32, and potential type 1 coherent feedforward loops on the right as Eomesa activates positive regulators of sox32 and potentially also vgll4l.

Results above suggest that Eomesa overexpression induces DFC fate based on ectopic expression of markers including *sox32* and *vgll4l* (Figures 2F, 5B,C). To resolve the conflict between the observed Eomesa-mediated repression of *vgll4l* expression at mid/late blastula stages (4 h.p.f.) but induction by early gastrulation (6 h.p.f.) we explored the role of Eomesa target Sox32 in *vgll4l* induction. We found that *sox32* overexpression through one-cell stage mRNA injection led to a dramatic upregulation of *vgll4l* expression in early gastrulas (6 h.p.f.; Figure 6A), suggesting localised Eomesa-mediated upregulation of *vgll4l* at the margin occurs through Sox32 rather than a switch in Eomesa function directly at the *vgll4l* locus. To test whether Sox32 is required for induction of *vgll4l* expression we performed knockdown using a previously validated and widely used antisense morpholino (Dickmeis et al., 2001). This knockdown clearly resulted in loss of DFC *vgll4l* expression (Figure 6B). Furthermore, *vgll4l* induction in locations outside the dorsal margin, caused by combinatorial *eomesa*/*mixl1*/*gata5* overexpression was also profoundly abrogated by Sox32 knockdown. Thus, during gastrula stages induction of *vgll4l* is via Sox32 rather than direct Eomesa activities.

*Vgll4l* expression was similarly upregulated outside the dorsal margin by combinatorial *mixl1*/*gata5* overexpression, and partially blocked by Sox32 KD (Figure 6B). However, *vgll4l* expression in *mixl1*/*gata5* overexpressing embryos was not as profoundly reduced on Sox32 KD as was the case for *eomesa*/*mixl1*/*gata5* overexpression. It is not completely clear whether this is because *mixl1*/*gata5* can activate *vgll4l* independent of Sox32 function, or due to the absence of Eomesa-mediated repression of *vgll4l.* Addition of *tbx16* to *mixl1*/*gata5* overexpression led to a significant increase in the fraction of embryos exhibiting ectopic *vgll4l* expression compared to *mixl1*/*gata5* alone (Figure 6B). We note that overexpression of *tbx16* alone was insufficient to enhance *vgll4l* expression in a significant fraction of embryos, and showed no ability to induce *sox32* expression (Figures 5B,C). It seems likely that while *tbx16* alone does not exert a strong influence at the *vgll4l* locus, *mixl1* and *gata5* can provide a context for *tbx16* to enhance *vgll4l* expression. This likely involves Tbx16 binding to *vgll4l* intron 1 (Figure 5D) rather than occurring via *sox32*, since Tbx16 cannot induce *sox32* expression, either individually or in combination with Mixl1 and Gata5 (Figures 4C, 5B).

Overall the present results combined with previous published reports from ourselves and others suggest a model wherein Eomesa acts within interlocking incoherent type 3 and coherent type 1 feedforward loops (Mangan and Alon, 2003) to repress *vgll4l* while combining with Nodal downstream effectors Mixl1 and Gata5 to activate *sox32*, which in turn activates *vgll4l* around the time of DFC specification. In addition to this, our analyses indicate that both mouse Eomes FL and ∆VR isoforms are functionally equivalent to Eomesa, suggesting phenotypic differences between zebrafish and mouse Eomes loss-of-function mutants are not likely to be driven by functional divergence, but rather redundancy with co-expressed factors in zebrafish such as Tbx16.

## Discussion

### Phenotypic differences between mouse and zebrafish Eomes loss-of-function mutants are not due to molecular divergence

T-box transcription factors are an ancient family of genes with many key roles in embryogenesis and disease. Lineage-specific differences that occurred in the family during vertebrate evolution have resulted in altered gene complements and diversity of splice isoforms in distinct evolutionary lineages (DeBenedittis and Jiao, 2011; Papaioannou, 2014). While AS events in specific evolutionary lineages have led to functional diversification of certain T-box factors, we have shown that Eomes loss-of-function phenotypic differences between mouse and zebrafish are unlikely to be due to evolutionary differences in Eomes protein function, but rather a degree of compensation by Tbx16 which is present in zebrafish but not placental mammals.

Though AS is an evolutionary means of increasing functional diversity within the proteome, our data suggests that Eomes exon 6 AS is not functionally important in the context of early development. In the case of the ∆VR splicing event a synonymous mutation created an alternative splice acceptor, however, our data suggests it is hardly used leading to majority production of the FL isoform. That the ∆VR isoform arose and is maintained in the tetrapod lineage may be due to substantial functional similarlity of FL and ∆VR isoforms, leading to a lack of selective pressure.

In overexpression studies the ∆VR isoform has the ability to induce trophoblast markers in embryonic stem cells (Niwa et al., 2005), and cardiac mesoderm markers in embryonal carcinoma cells (Costello et al., 2011). Moreover, both FL and ∆VR isoforms can induce organizer and DFC markers while repressing ectoderm markers on overexpression in zebrafish. These observations provide further evidence of their functional equivalence.

The present data demonstrate that the ∆VR and ∆CTD isoforms are only weakly expressed compared with FL Eomes. While we find no evidence for the functional importance of the VR it is intriguing that it is both highly conserved and known to be phosphorylated (Huttlin et al., 2010). It is possible that these isoforms potentially make substantial contributions in contexts we have not explored. The present evidence, however, suggests that in mice, as in zebrafish the FL isoform is the more important molecule.

Given the complexities of mouse Eomes mutant phenotypes it would be interesting to explore isoform-specific functions in mouse. This could be achieved through either modifying the endogenous Eomes locus in embryonic stem cells (ESCs) such that only specific isoforms could be expressed, or using isoform-specific inducible transgenes in Eomes null mutant ESCs. This could be combined with directed differentiation procedures to determine whether there are detectable isoform-specific functions in relevant cell types. Whether zebrafish Eomesa, or other T-box factors can functionally substitute for mouse Eomes could also be tested in a similar system. Alternatively, the genetically modified ESCs could be used to make mice in an attempt to study isoform and orthologue functions *in vivo*.

### Functional similarities and differences of Eomesa and Tbx16

We previously demonstrated that Eomesa and Tbx16 display overlapping genomic binding profiles in early zebrafish embryos (Nelson et al., 2017). Whether they are functionally redundant, however, had not been explored. The present experiments strongly suggest that Eomesa and Tbx16 redundantly regulate the homeodomain transcription factor *mixl1*, which has key conserved functions in endoderm formation in zebrafish and mouse (Kikuchi et al., 2000; Hart et al., 2002). *Mixl1* mutants suffer profound loss of endoderm (Kikuchi et al., 2000). Reduced expression of *mixl1* in the margin of late blastulas/early gastrulas is coupled with reduced numbers of endoderm progenitors in late gastrulation in both MZ*eomesa* mutants, and on *tbx16* knockdown (Du et al., 2012; Nelson et al., 2017). It therefore seems likely that the enhanced reduction of *mixl1* expression on *tbx16* knockdown in *eomesa* mutants in the present study would lead to increased loss of endoderm progenitors. Our previous RNA-seq analyses indicate that expression of *tbx16* is not significantly different in MZ*eomesa* mutants (Nelson et al., 2014). It therefore seems likely that Tbx16 partially compensates for loss of Eomesa during zebrafish endoderm formation. Our study therefore highlights a consistent requirement for T-box function in vertebrate endoderm formation. Interestingly, while multiple orthologous T-box factors have similar expression domains in early zebrafish and mouse embryogenesis, those domains are typically expanded in zebrafish (Wardle and Papaioannou, 2008). Coupled with its rapid rate of development and the greater number of T-box factors in zebrafish, there is likely to be a higher degree of T-box factor co-expression, enhancing the probability of redundancy.

While Eomesa and Tbx16 share some redundant functions we also identified key differences. It was previously shown that Eomesa can combine with Mixl1 and Gata5 to drive expression of *sox32* at the animal pole (Bjornson et al., 2005). However, Tbx16 does not appear to have the same ability as Eomesa to drive *sox32* expression either individually or in combination with Mixl1 and Gata5, even though *sox32* expressing cells appear to exhibit *tbx16* expression in single-cell RNA-seq data. This is consistent with previous observations that M*eomesa* and MZ*eomesa* mutants have reduced expression of *sox32* during gastrulation without complete loss (Du et al., 2012; Xing et al., 2022). It therefore seems likely that Tbx16 is sufficient to rescue certain Eomesa functions but cannot completely compensate for its loss. It is further notable that Tbx16 does not seem individually able to induce the dorsal mesoderm marker *noto* as Eomesa can. However, given that Eomesa acts upstream of Nodal (Xu et al., 2014; Xing et al., 2022) it seems likely that major differences in outcome between *eomesa* and *tbx16* overexpression stem from enhanced Nodal signalling on *eomesa* overexpression. It will be interesting to learn more about the common and unique functional activities of Eomes and Tbx16 that drive target gene expression.

Eomesa and Tbx16 are only distantly related within the T-box family (25.3% of Tbx16 amino acid identity), with the majority of conserved amino acids occurring within the T-box domain. Whether they are likely to act in similar protein complexes to regulate their target genes is therefore unclear. A key study in *Xenopus*, however, suggested the specificity of target gene induction is primarily mediated by the T-box itself, rather than NTDs and CTDs (Conlon et al., 2001). The same study demonstrated a single asparagine to lysine substitution in *Xenopus* Eomes and VegT T-box domains, alter their inductive properties to mimic Brachyury (Conlon et al., 2001). Importantly, both Eomesa and Tbx16 (which has been proposed as the zebrafish orthologue of *Xenopus VegT* (Griffin et al., 1998)) share the same critical asparagine. Our data suggest that the N320K mutation has little effect on induction of Eomesa target genes explored here, is unlikely to prevent T-box interaction with co-factors Mixl1 and Gata5, or substantially account for differences with Tbxta in endoderm and DFC formation. In fact, analysis of single-cell RNA-seq data suggests that the greater importance of Eomesa and Tbx16 in endoderm formation is more likely to be attributable to lesser Tbxta expression in the endoderm. It is therefore possible that Eomesa and Tbx16 also have overlapping roles in endoderm formation downstream of driving *mixl1* expression in presumptive endoderm that are yet to be elucidated.

Interestingly, participation of zebrafish Tbx16 in processes controlled by Eomes in mice is not limited to endoderm formation. For example, while Eomes acts upstream of basic helix-loop-helix transcription factor gene *Mesp1* to specify cardiac mesoderm in mice (Costello et al., 2011), Tbx16 regulates the orthologous gene *mespaa* in zebrafish (Garnett et al., 2009). Eomes loss-of-function leads to aberrant mesoderm cell migration during mouse gastrulation, while *tbx16* zebrafish mutants also exhibit cell-autonomous defects in mesoderm migration (Ho and Kane, 1990; Arnold et al., 2008a). Remarkably, potentially interesting parallels continue to emerge, such as the requirements for zebrafish Tbx16 and mouse Eomes in blood progenitors (Rohde et al., 2004; Harland et al., 2021). It is therefore possible that the presence of Tbx16 in teleost fish has led to a reduced requirement for Eomes in multiple developmental contexts.

The present study focuses on early embryonic development, however, Eomes is known to have later roles in neurological development, as well as in the immune system. Importantly, Eomes is an key regulator of neurogenesis in the subventricular zone, and loss leads to microcephaly and severe behavioural defects (Arnold et al., 2008b). Though *eomesa* is equivalently expressed in the telencephalon of developing zebrafish larvae, whether null mutants have an equivalent phenotype is unknown (Mione et al., 2001; Du et al., 2012). If they do not, however, it is unlikely to be due to redundancy with *tbx16*, which is absent from the developing brain. Similarly, it is unclear whether *eomesa* mutants exhibit defects in the immune system, such as in T cell differentiation and NK cell development and function as in mammals (Simonetta et al., 2016; D'Cruz et al., 2009). Both *eomesa* and *eomesb* are co-expressed in lymphocytes in fish, however, suggesting they may be redundant in the immune system (Takizawa et al., 2007; Takizawa et al., 2014).

While T-box factor redundancy during development is not a novel concept e.g. (Amacher et al., 2002; Garnett et al., 2009; Jahangiri et al., 2012; Gentsch et al., 2013; Nelson et al., 2017), the molecular basis for this redundancy (or indeed T-box factor molecular interactions in general) is not well understood. In future it will be interesting to study whether redundant T-box factors recruit similar co-factors to regulate gene expression, and whether this occurs through conserved or divergent amino acid sequences and structural motifs.

### On the roles of Eomesa, Tbx16 and Tbxta in dorsal mesoderm

While Eomesa is capable of inducing dorsal mesoderm markers such as *noto* and *chrd* in zebrafish embryos, it is notable that their expression is normal in the absence of Eomesa (Bruce et al., 2003; Du et al., 2012). However, it is also notable that *tbxta* and *tbx16* do not show altered expression in *eomesa* mutants in published WISH and RNA-seq datasets (Du et al., 2012; Nelson et al., 2014). It is therefore possible that Tbx16 and Tbxta are amongst factors compensating for the loss of Eomesa. In support of this, Tbxta has been shown to directly activate *noto* expression, and *tbxta* mutants fail to maintain *noto* expression in mid/late gastrulation stages, leading to loss of notochord (Melby et al., 1997; Morley et al., 2009). Similarly, Tbx16 is required to maintain *chrd* expression in axial structures at mid/late gastrulation stages (Miller-Bertoglio et al., 1997; Warga et al., 2013). The reduced expression of both *noto* in *tbxta* mutants and *chrd* in *tbx16* mutants follows the decline in *eomesa* mRNA expression levels, suggesting that Tbxta and Tbx16 maintain the expression of dorsal mesoderm markers in the absence of Eomesa. Nevertheless, our results indicate that of these T-box factors, only Eomesa is sufficient to induce ectopic dorsal mesoderm marker expression. This suggests key differences in the molecular functions of these T-box factors. It is possible that co-factors required for dorsal mesoderm induction by Eomesa are localised throughout the margin while those required by Tbxta and Tbx16 are restricted to the dorsal margin. Alternatively, given Eomesa regulates expression of Nodal pathway ligands which are required for dorsal mesoderm fates, it is possible that Eomesa but not Tbxta/Tbx16 is capable of expanding dorsal mesoderm through upregulation of Nodal signalling (Du et al., 2012; Xu et al., 2014; Xing et al., 2022).

### Eomesa, Tbx16, Mixl1 and Gata5 activities during dorsal forerunner cell formation

Loss of Eomesa leads to upregulation of *vgll4l* during blastula stages whereas overexpression of *eomesa* causes repression of *vgll4l* (Nelson et al., 2014). The present experiments suggests that Eomesa acts within feedforward loops to repress *vgll4l* expression until activators including Sox32 accumulate to drive *vgll4l* in DFCs at the onset of gastrulation. Given that Eomesa is maternally contributed and not spatially restricted in early development (Du et al., 2012), while accumulation of *vgll4l* activators is principally driven by Nodal at the dorsal margin, this suggests a model wherein Eomesa controls the specificity and timing of *vgll4l* induction. *Eomesa* mRNA steadily declines during blastula stages as expression of *mixl1*, *gata5*, *tbx16* and *sox32* increase, and is virtually undetectable at the onset of gastrulation, (Figure 3A and Bruce et al., 2003; Figiel et al., 2021). While Eomesa protein does persist through gastrulation (Du et al., 2012) it seems likely that temporal and spatial changes in abundance of *vgll4l* activators and repressors acting within these feedforward loops cooperatively regulate the specificity of *vgll4l* expression during DFC specification.

Genetic data, however, suggest that our model is likely to be incomplete. While Sox32 is required for correct DFC formation (Alexander et al., 1999; Essner et al., 2005), upstream regulators *mixl1* and *gata5* are not required for DFC formation individually or in combination. Rather *mixl1* and *gata5* seem to be strictly required upstream of *sox32* for correct endoderm formation (Reiter et al., 1999; Kikuchi et al., 2000; Reiter et al., 2001). While we cannot discount the possibility of *mixl1* and *gata5* expression in precursors of DFCs, present evidence suggests that there are either alternative upstream regulators of *sox32* in DFCs vs. endoderm, or additional redundant factors in DFCs rescuing the requirement for *mixl1* and *gata5*. However, given the apparent requirement for Nodal signalling in DFC formation (Alexander and Stainier, 1999), it seems likely that whatever the upstream regulators of *sox32* expression in DFCs they will be Nodal-dependent. Overall this highlights a lack of understanding of the gene regulatory networks that direct DFC vs. endoderm formation, which will be a key focus of our future work.

Recent evidence suggests Vgll4l is required for *tbx16* expression during DFC formation (Fillatre et al., 2019). That Tbx16 binds the *vgll4l* promoter during gastrulation could suggest that complex regulatory loops control DFC formation and maintenance. The ability of Eomesa to induce ectopic DFCs during early gastrulation combined with expression of mouse Eomes in progenitors of the node and requirement for correct node formation (Arnold et al., 2008a; Costello et al., 2011) suggests the potential for a conserved role in establishment of left-right asymmetry with some degree of redundancy with Tbx16 in zebrafish. However, a role for the *vgll4l* mammalian homologue *Vgll4* in left-right asymmetry has yet to be determined. Further study of the mechanistic parallels in T-box factor mediated formation of zebrafish DFCs and mouse node would be beneficial to gain a more detailed evo-devo understanding of this process.

The diversity of DFC marker gene induction observed in this study was particularly striking, and points to dorsally localised determinants of DFC identity and function that are less readily induced by Eomesa and Eomes. We found that between them Eomesa and mouse Eomes isoforms were able to induce *sox32, sox17, vgll4l* and *foxj1a*. *Sox32* is required for maintenance of DFC identify and formation of the left-right organiser (Alexander et al., 1999; Essner et al., 2005) while its downstream target *sox17* is required for correct left-right organiser function (Aamar and Dawid, 2010). *Vgll4l* is a key mediator of Hippo signalling and regulates epigenetic programming of DFC by controlling the expression of writers and readers of DNA methylation, influencing DFC proliferation, apoptosis and ciliagenesis (Fillatre et al., 2019). *Foxj1a* is the master regulator if motile cilia formation (Yu et al., 2008). That the Eomes-mediated induction of *foxj1a* was more restricted to the dorsal margin than that of other markers suggests that Vgll4l and the Sox and T-box factors known to be involved in DFC formation are not sufficient to fully induce DFC identity. Other localised cues (physical, mechanical, signalling or cell intrinsic factors) are therefore likely to be involved in *foxj1a* induction.

Overall we conclude that enhanced AS in mammals has not significantly altered Eomes function in early embryogenesis. Rather we conclude that the different degrees of T-box factor co-expression and the presence/absence of additional factors including Tbx16 has modulated the severity of the Eomes null mutant phenotype in the embryo proper between mouse and zebrafish. Furthermore, we conclude that in zebrafish Eomesa participates in DFC formation through directing feedforward loops via *sox32* to control *vgll4l* expression. Our results therefore provide novel insights into evolutionary differences in vertebrate endoderm formation, and the gene regulatory networks involved in controlling the zebrafish left-right organiser formation.

## Data Availability

The datasets presented in this study can be found in online repositories. The names of the repository/repositories and accession number(s) can be found below: The single-cell RNA-seq data used in the study is from a published paper, and is available in NCBI GEO (accession GSM3067190).

## References

[B1] Ashe H. L. Monks J. Wijgerde M. Fraser P. Proudfoot N. J. (1997). Intergenic transcription and transinduction of the human beta-globin locus. Genes Dev. 11, 2494–2509. 10.1101/gad.11.19.2494 9334315 PMC316561

[B2] Aamar E. Dawid I. B. (2010). Sox17 and chordin are required for formation of Kupffer's vesicle and left-right asymmetry determination in zebrafish. Dev. Dyn. 239, 2980–2988. 10.1002/dvdy.22431 20925124 PMC3090657

[B3] Adams S. E. Johnson I. D. Braddock M. Kingsman A. J. Kingsman S. M. Edwards R. M. (1988). Synthesis of a gene for the HIV transactivator protein TAT by a novel single stranded approach involving *in vivo* gap repair. Nucleic Acids Res. 16, 4287–4298. 10.1093/nar/16.10.4287 3288969 PMC336630

[B4] Ahn D. You K. H. Kim C. H. (2012). Evolution of the tbx6/16 subfamily genes in vertebrates: Insights from zebrafish. Mol. Biol. Evol. 29, 3959–3983. 10.1093/molbev/mss199 22915831

[B5] Alexander J. Rothenberg M. Henry G. L. Stainier D. Y. (1999). Casanova plays an early and essential role in endoderm formation in zebrafish. Dev. Biol. 215, 343–357. 10.1006/dbio.1999.9441 10545242

[B6] Alexander J. Stainier D. Y. (1999). A molecular pathway leading to endoderm formation in zebrafish. Curr. Biol. 9, 1147–1157. 10.1016/S0960-9822(00)80016-0 10531029

[B7] Amacher S. L. Draper B. W. Summers B. R. Kimmel C. B. (2002). The zebrafish T-box genes no tail and spadetail are required for development of trunk and tail mesoderm and medial floor plate. Development 129, 3311–3323. 10.1242/dev.129.14.3311 12091302

[B8] Arnold S. J. Hofmann U. K. Bikoff E. K. Robertson E. J. (2008a). Pivotal roles for eomesodermin during axis formation, epithelium-to-mesenchyme transition and endoderm specification in the mouse. Development 135, 501–511. 10.1242/dev.014357 18171685 PMC7116389

[B9] Arnold S. J. Huang G. J. Cheung A. F. Era T. Nishikawa S. Bikoff E. K. (2008b). The T-box transcription factor Eomes/Tbr2 regulates neurogenesis in the cortical subventricular zone. Genes Dev. 22, 2479–2484. 10.1101/gad.475408 18794345 PMC2546697

[B10] Barrett T. Wilhite S. E. Ledoux P. Evangelista C. Kim I. F. Tomashevsky M. (2013). NCBI GEO: Archive for functional genomics data sets--update. Nucleic Acids Res. 41, D991–D995. 10.1093/nar/gks1193 23193258 PMC3531084

[B11] Bisgrove B. W. Snarr B. S. Emrazian A. Yost H. J. (2005). Polaris and Polycystin-2 in dorsal forerunner cells and Kupffer's vesicle are required for specification of the zebrafish left-right axis. Dev. Biol. 287, 274–288. 10.1016/j.ydbio.2005.08.047 16216239

[B12] Bjornson C. R. Griffin K. J. Farr G. H., 3R. D. Terashima A. Himeda C. Kikuchi Y. (2005). Eomesodermin is a localized maternal determinant required for endoderm induction in zebrafish. Dev. Cell 9, 523–533. 10.1016/j.devcel.2005.08.010 16198294

[B13] Bogdanovic O. Fernandez-Minan A. Tena J. J. De La Calle-Mustienes E. Hidalgo C. Van Kruysbergen I. (2012). Dynamics of enhancer chromatin signatures mark the transition from pluripotency to cell specification during embryogenesis. Genome Res. 22, 2043–2053. 10.1101/gr.134833.111 22593555 PMC3460198

[B14] Bruce A. E. Howley C. Zhou Y. Vickers S. L. Silver L. M. King M. L. (2003). The maternally expressed zebrafish T-box gene eomesodermin regulates organizer formation. Development 130, 5503–5517. 10.1242/dev.00763 14530296

[B15] Conlon F. L. Fairclough L. Price B. M. Casey E. S. Smith J. C. (2001). Determinants of T box protein specificity. Development 128, 3749–3758. 10.1242/dev.128.19.3749 11585801

[B16] Costello I. Pimeisl I. M. Drager S. Bikoff E. K. Robertson E. J. Arnold S. J. (2011). The T-box transcription factor Eomesodermin acts upstream of Mesp1 to specify cardiac mesoderm during mouse gastrulation. Nat. Cell Biol. 13, 1084–1091. 10.1038/ncb2304 21822279 PMC4531310

[B17] Crooks G. E. Hon G. Chandonia J. M. Brenner S. E. (2004). WebLogo: A sequence logo generator. Genome Res. 14, 1188–1190. 10.1101/gr.849004 15173120 PMC419797

[B18] D'Cruz L. M. Rubinstein M. P. Goldrath A. W. (2009). Surviving the crash: Transitioning from effector to memory CD8+ T cell. Semin. Immunol. 21, 92–98. 10.1016/j.smim.2009.02.002 19269192 PMC2671236

[B19] Debenedittis P. Jiao K. (2011). Alternative splicing of T-box transcription factor genes. Biochem. Biophys. Res. Commun. 412, 513–517. 10.1016/j.bbrc.2011.08.010 21856288 PMC3171624

[B20] Dickmeis T. Mourrain P. Saint-Etienne L. Fischer N. Aanstad P. Clark M. (2001). A crucial component of the endoderm formation pathway, CASANOVA, is encoded by a novel sox-related gene. Genes Dev. 15, 1487–1492. 10.1101/gad.196901 11410529 PMC312720

[B21] Du S. Draper B. W. Mione M. Moens C. B. Bruce A. (2012). Differential regulation of epiboly initiation and progression by zebrafish Eomesodermin A. Dev. Biol. 362, 11–23. 10.1016/j.ydbio.2011.10.036 22142964 PMC3259739

[B22] Eggermont J. Proudfoot N. J. (1993). Poly(A) signals and transcriptional pause sites combine to prevent interference between RNA polymerase II promoters. EMBO J. 12, 2539–2548. 10.1002/j.1460-2075.1993.tb05909.x 8508777 PMC413492

[B23] Essner J. J. Amack J. D. Nyholm M. K. Harris E. B. Yost H. J. (2005). Kupffer's vesicle is a ciliated organ of asymmetry in the zebrafish embryo that initiates left-right development of the brain, heart and gut. Development 132, 1247–1260. 10.1242/dev.01663 15716348

[B24] Faial T. Bernardo A. S. Mendjan S. Diamanti E. Ortmann D. Gentsch G. E. (2015). Brachyury and SMAD signalling collaboratively orchestrate distinct mesoderm and endoderm gene regulatory networks in differentiating human embryonic stem cells. Development 142, 2121–2135. 10.1242/dev.117838 26015544 PMC4483767

[B25] Figiel D. M. Elsayed R. Nelson A. C. (2021). Investigating the molecular guts of endoderm formation using zebrafish. Brief. Funct. Genomics 20, 394–406. 10.1093/bfgp/elab013 33754635

[B26] Fillatre J. Fauny J. D. Fels J. A. Li C. Goll M. Thisse C. (2019). TEADs, Yap, Taz, Vgll4s transcription factors control the establishment of Left-Right asymmetry in zebrafish. Elife 8, e45241. 10.7554/eLife.45241 31513014 PMC6759317

[B27] Fornes O. Castro-Mondragon J. A. Khan A. Van Der Lee R. Zhang X. Richmond P. A. (2020). Jaspar 2020: Update of the open-access database of transcription factor binding profiles. Nucleic Acids Res. 48, D87–D92. 10.1093/nar/gkz1001 31701148 PMC7145627

[B28] Garnett A. T. Han T. M. Gilchrist M. J. Smith J. C. Eisen M. B. Wardle F. C. (2009). Identification of direct T-box target genes in the developing zebrafish mesoderm. Development 136, 749–760. 10.1242/dev.024703 19158186 PMC2685943

[B29] Gentsch G. E. Monteiro R. S. Smith J. C. (2017). Cooperation between T-box factors regulates the continuous segregation of germ layers during vertebrate embryogenesis. Curr. Top. Dev. Biol. 122, 117–159. 10.1016/bs.ctdb.2016.07.012 28057262

[B30] Gentsch G. E. Owens N. D. Martin S. R. Piccinelli P. Faial T. Trotter M. W. (2013). *In vivo* T-box transcription factor profiling reveals joint regulation of embryonic neuromesodermal bipotency. Cell Rep. 4, 1185–1196. 10.1016/j.celrep.2013.08.012 24055059 PMC3791401

[B31] Glasauer S. M. K. Neuhauss S. C. F. (2014). Whole-genome duplication in teleost fishes and its evolutionary consequences. Mol. Genet. Genomics 289, 1045–1060. 10.1007/s00438-014-0889-2 25092473

[B32] Goujon M. Mcwilliam H. Li W. Valentin F. Squizzato S. Paern J. (2010). A new bioinformatics analysis tools framework at EMBL-EBI. Nucleic Acids Res. 38, W695–W699. 10.1093/nar/gkq313 20439314 PMC2896090

[B33] Griffin K. J. Amacher S. L. Kimmel C. B. Kimelman D. (1998). Molecular identification of spadetail: Regulation of zebrafish trunk and tail mesoderm formation by T-box genes. Development 125, 3379–3388. 10.1242/dev.125.17.3379 9693141

[B34] Grinblat Y. Sive H. (2001). Zic Gene expression marks anteroposterior pattern in the presumptive neurectoderm of the zebrafish gastrula. Dev. Dyn. 222, 688–693. 10.1002/dvdy.1221 11748837

[B35] Hafemeister C. Satija R. (2019). Normalization and variance stabilization of single-cell RNA-seq data using regularized negative binomial regression. Genome Biol. 20, 296. 10.1186/s13059-019-1874-1 31870423 PMC6927181

[B36] Harland L. T. G. Simon C. S. Senft A. D. Costello I. Greder L. Imaz-Rosshandler I. (2021). The T-box transcription factor Eomesodermin governs haemogenic competence of yolk sac mesodermal progenitors. Nat. Cell Biol. 23, 61–74. 10.1038/s41556-020-00611-8 33420489 PMC7610381

[B37] Hart A. H. Hartley L. Sourris K. Stadler E. S. Li R. Stanley E. G. (2002). Mixl1 is required for axial mesendoderm morphogenesis and patterning in the murine embryo. Development 129, 3597–3608. 10.1242/dev.129.15.3597 12117810

[B38] Ho R. K. Kane D. A. (1990). Cell-autonomous action of zebrafish spt-1 mutation in specific mesodermal precursors. Nature 348, 728–730. 10.1038/348728a0 2259382

[B39] Huttlin E. L. Jedrychowski M. P. Elias J. E. Goswami T. Rad R. Beausoleil S. A. (2010). A tissue-specific atlas of mouse protein phosphorylation and expression. Cell 143, 1174–1189. 10.1016/j.cell.2010.12.001 21183079 PMC3035969

[B40] Jahangiri L. Nelson A. C. Wardle F. C. (2012). A cis-regulatory module upstream of deltaC regulated by Ntla and Tbx16 drives expression in the tailbud, presomitic mesoderm and somites. Dev. Biol. 371, 110–120. 10.1016/j.ydbio.2012.07.002 22877946 PMC3460241

[B41] Jowett T. Lettice L. (1994). Whole-mount *in situ* hybridizations on zebrafish embryos using a mixture of digoxigenin- and fluorescein-labelled probes. Trends Genet. 10, 73–74. 10.1016/0168-9525(94)90220-8 8178366

[B42] Karolchik D. Hinrichs A. S. Furey T. S. Roskin K. M. Sugnet C. W. Haussler D. (2004). The UCSC Table Browser data retrieval tool. Nucleic Acids Res. 32, D493–D496. 10.1093/nar/gkh103 14681465 PMC308837

[B43] Keren H. Lev-Maor G. Ast G. (2010). Alternative splicing and evolution: Diversification, exon definition and function. Nat. Rev. Genet. 11, 345–355. 10.1038/nrg2776 20376054

[B44] Kikuchi Y. Trinh L. A. Reiter J. F. Alexander J. Yelon D. Stainier D. Y. (2000). The zebrafish bonnie and clyde gene encodes a Mix family homeodomain protein that regulates the generation of endodermal precursors. Genes Dev. 14, 1279–1289. 10.1101/gad.14.10.1279 10817762 PMC316618

[B45] Kimmel C. B. Ballard W. W. Kimmel S. R. Ullmann B. Schilling T. F. (1995). Stages of embryonic development of the zebrafish. Dev. Dyn. 203, 253–310. 10.1002/aja.1002030302 8589427

[B46] Lolas M. Valenzuela P. D. Tjian R. Liu Z. (2014). Charting Brachyury-mediated developmental pathways during early mouse embryogenesis. Proc. Natl. Acad. Sci. U. S. A. 111, 4478–4483. 10.1073/pnas.1402612111 24616493 PMC3970479

[B47] Mangan S. Alon U. (2003). Structure and function of the feed-forward loop network motif. Proc. Natl. Acad. Sci. U. S. A. 100, 11980–11985. 10.1073/pnas.2133841100 14530388 PMC218699

[B48] Marcellini S. Technau U. Smith J. C. Lemaire P. (2003). Evolution of brachyury proteins: Identification of a novel regulatory domain conserved within bilateria. Dev. Biol. 260, 352–361. 10.1016/s0012-1606(03)00244-6 12921737

[B49] Mcwilliam H. Li W. Uludag M. Squizzato S. Park Y. M. Buso N. (2013). Analysis tool web services from the EMBL-EBI. Nucleic Acids Res. 41, W597–W600. 10.1093/nar/gkt376 23671338 PMC3692137

[B50] Melby A. E. Kimelman D. Kimmel C. B. (1997). Spatial regulation of floating head expression in the developing notochord. Dev. Dyn. 209, 156–165. 10.1002/(SICI)1097-0177(199706)209:2<156::AID-AJA2>3.0.CO;2-H 9186051

[B51] Melville M. Lun A. Djekidel M. N. Hao Y. (2020). uwot: The uniform manifold approximation and projection (UMAP) method for dimensionality reduction. R package version 0.1.11. Available at: https://cran.r-project.org/web/packages/uwot/index.html .

[B52] Miller-bertoglio V. E. Fisher S. Sanchez A. Mullins M. C. Halpern M. E. (1997). Differential regulation of chordin expression domains in mutant zebrafish. Dev. Biol. 192, 537–550. 10.1006/dbio.1997.8788 9441687

[B53] Mione M. Shanmugalingam S. Kimelman D. Griffin K. (2001). Overlapping expression of zebrafish T-brain-1 and eomesodermin during forebrain development. Mech. Dev. 100, 93–97. 10.1016/s0925-4773(00)00501-3 11118891

[B54] Morley R. H. Lachani K. Keefe D. Gilchrist M. J. Flicek P. Smith J. C. (2009). A gene regulatory network directed by zebrafish No tail accounts for its roles in mesoderm formation. Proc. Natl. Acad. Sci. U. S. A. 106, 3829–3834. 10.1073/pnas.0808382106 19225104 PMC2656165

[B55] Nelson A. C. Cutty S. J. Gasiunas S. N. Deplae I. Stemple D. L. Wardle F. C. (2017). *In vivo* regulation of the zebrafish endoderm progenitor niche by T-box transcription factors. Cell Rep. 19, 2782–2795. 10.1016/j.celrep.2017.06.011 28658625 PMC5494305

[B56] Nelson A. C. Cutty S. J. Niini M. Stemple D. L. Flicek P. Houart C. (2014). Global identification of Smad2 and Eomesodermin targets in zebrafish identifies a conserved transcriptional network in mesendoderm and a novel role for Eomesodermin in repression of ectodermal gene expression. BMC Biol. 12, 81. 10.1186/s12915-014-0081-5 25277163 PMC4206766

[B57] Nelson A. C. Pillay N. Henderson S. Presneau N. Tirabosco R. Halai D. (2012). An integrated functional genomics approach identifies the regulatory network directed by brachyury (T) in chordoma. J. Pathol. 228, 274–285. 10.1002/path.4082 22847733 PMC6089345

[B58] Niwa H. Toyooka Y. Shimosato D. Strumpf D. Takahashi K. Yagi R. (2005). Interaction between Oct3/4 and Cdx2 determines trophectoderm differentiation. Cell 123, 917–929. 10.1016/j.cell.2005.08.040 16325584

[B59] Nowotschin S. Costello I. Piliszek A. Kwon G. S. Mao C. A. Klein W. H. (2013). The T-box transcription factor Eomesodermin is essential for AVE induction in the mouse embryo. Genes Dev. 27, 997–1002. 10.1101/gad.215152.113 23651855 PMC3656330

[B60] Papaioannou V. E. (2014). The T-box gene family: Emerging roles in development, stem cells and cancer. Development 141, 3819–3833. 10.1242/dev.104471 25294936 PMC4197708

[B61] Pearce E. L. Mullen A. C. Martins G. A. Krawczyk C. M. Hutchins A. S. Zediak V. P. (2003). Control of effector CD8(+) T cell function by the transcription factor Eomesodermin. Science 302, 1041–1043. 10.1126/science.1090148 14605368

[B62] Picozzi P. Wang F. Cronk K. Ryan K. (2009). Eomesodermin requires transforming growth factor-beta/activin signaling and binds Smad2 to activate mesodermal genes. J. Biol. Chem. 284, 2397–2408. 10.1074/jbc.M808704200 19036723 PMC2629102

[B63] Probst S. Arnold S. J. (2017). Eomesodermin-at dawn of cell fate decisions during early embryogenesis. Curr. Top. Dev. Biol. 122, 93–115. 10.1016/bs.ctdb.2016.09.001 28057273

[B64] Ramani A. K. Calarco J. A. Pan Q. Mavandadi S. Wang Y. Nelson A. C. (2011). Genome-wide analysis of alternative splicing in *Caenorhabditis elegans* . Genome Res. 21, 342–348. 10.1101/gr.114645.110 21177968 PMC3032936

[B65] Reiter J. F. Alexander J. Rodaway A. Yelon D. Patient R. Holder N. (1999). Gata5 is required for the development of the heart and endoderm in zebrafish. Genes Dev. 13, 2983–2995. 10.1101/gad.13.22.2983 10580005 PMC317161

[B66] Reiter J. F. Kikuchi Y. Stainier D. Y. (2001). Multiple roles for Gata5 in zebrafish endoderm formation. Development 128, 125–135. 10.1242/dev.128.1.125 11092818

[B67] Rohde L. A. Oates A. C. Ho R. K. (2004). A crucial interaction between embryonic red blood cell progenitors and paraxial mesoderm revealed in spadetail embryos. Dev. Cell 7, 251–262. 10.1016/j.devcel.2004.07.010 15296721 PMC2801434

[B68] Russ A. P. Wattler S. Colledge W. H. Aparicio S. A. Carlton M. B. Pearce J. J. (2000). Eomesodermin is required for mouse trophoblast development and mesoderm formation. Nature 404, 95–99. 10.1038/35003601 10716450

[B69] Sebe-pedros A. Ariza-Cosano A. Weirauch M. T. Leininger S. Yang A. Torruella G. (2013). Early evolution of the T-box transcription factor family. Proc. Natl. Acad. Sci. U. S. A. 110, 16050–16055. 10.1073/pnas.1309748110 24043797 PMC3791752

[B70] Sievers F. Wilm A. Dineen D. Gibson T. J. Karplus K. Li W. (2011). Fast, scalable generation of high-quality protein multiple sequence alignments using Clustal Omega. Mol. Syst. Biol. 7, 539. 10.1038/msb.2011.75 21988835 PMC3261699

[B71] Simonetta F. Pradier A. Roosnek E. (2016). T-Bet and eomesodermin in NK cell development, maturation, and function. Front. Immunol. 7, 241. 10.3389/fimmu.2016.00241 27379101 PMC4913100

[B72] Speir M. L. Zweig A. S. Rosenbloom K. R. Raney B. J. Paten B. Nejad P. (2016). The UCSC genome browser database: 2016 update. Nucleic Acids Res. 44, D717–D725. 10.1093/nar/gkv1275 26590259 PMC4702902

[B73] Strumpf D. Mao C. A. Yamanaka Y. Ralston A. Chawengsaksophak K. Beck F. (2005). Cdx2 is required for correct cell fate specification and differentiation of trophectoderm in the mouse blastocyst. Development 132, 2093–2102. 10.1242/dev.01801 15788452

[B74] Stuart T. Butler A. Hoffman P. Hafemeister C. Papalexi E. Mauck W. M. (2019). Comprehensive integration of single-cell data. Cell 177, 1888–1902. 10.1016/j.cell.2019.05.031 31178118 PMC6687398

[B75] Takizawa F. Araki K. Ito K. Moritomo T. Nakanishi T. (2007). Expression analysis of two Eomesodermin homologues in zebrafish lymphoid tissues and cells. Mol. Immunol. 44, 2324–2331. 10.1016/j.molimm.2006.11.018 17194477

[B76] Takizawa F. Araki K. Ohtani M. Toda H. Saito Y. Lampe V. S. (2014). Transcription analysis of two Eomesodermin genes in lymphocyte subsets of two teleost species. Fish. Shellfish Immunol. 36, 215–222. 10.1016/j.fsi.2013.11.004 24239596

[B77] Talbot W. S. Trevarrow B. Halpern M. E. Melby A. E. Farr G. Postlethwait J. H. (1995). A homeobox gene essential for zebrafish notochord development. Nature 378, 150–157. 10.1038/378150a0 7477317

[B78] Teo A. K. Arnold S. J. Trotter M. W. Brown S. Ang L. T. Chng Z. (2011). Pluripotency factors regulate definitive endoderm specification through eomesodermin. Genes Dev. 25, 238–250. 10.1101/gad.607311 21245162 PMC3034899

[B79] Tsankov A. M. Gu H. Akopian V. Ziller M. J. Donaghey J. Amit I. (2015). Transcription factor binding dynamics during human ES cell differentiation. Nature 518, 344–349. 10.1038/nature14233 25693565 PMC4499331

[B80] Vesterlund L. Jiao H. Unneberg P. Hovatta O. Kere J. (2011). The zebrafish transcriptome during early development. BMC Dev. Biol. 11, 30. 10.1186/1471-213X-11-30 21609443 PMC3118190

[B81] Wagner D. E. Weinreb C. Collins Z. M. Briggs J. A. Megason S. G. Klein A. M. (2018). Single-cell mapping of gene expression landscapes and lineage in the zebrafish embryo. Science 360, 981–987. 10.1126/science.aar4362 29700229 PMC6083445

[B82] Wang E. T. Sandberg R. Luo S. Khrebtukova I. Zhang L. Mayr C. (2008). Alternative isoform regulation in human tissue transcriptomes. Nature 456, 470–476. 10.1038/nature07509 18978772 PMC2593745

[B83] Wardle F. C. Papaioannou V. E. (2008). Teasing out T-box targets in early mesoderm. Curr. Opin. Genet. Dev. 18, 418–425. 10.1016/j.gde.2008.07.017 18778771 PMC2700021

[B84] Warga R. M. Mueller R. L. Ho R. K. Kane D. A. (2013). Zebrafish Tbx16 regulates intermediate mesoderm cell fate by attenuating Fgf activity. Dev. Biol. 383, 75–89. 10.1016/j.ydbio.2013.08.018 24008197 PMC3919442

[B85] Waterhouse A. M. Procter J. B. Martin D. M. Clamp M. Barton G. J. (2009). Jalview Version 2--a multiple sequence alignment editor and analysis workbench. Bioinformatics 25, 1189–1191. 10.1093/bioinformatics/btp033 19151095 PMC2672624

[B86] Westerfield M. (2000). The zebrafish book. A guide for the laboratory use of zebrafish (*Danio rerio*). 4th Ed. Eugene: University of Oregon Press.

[B87] White R. J. Collins J. E. Sealy I. M. Wali N. Dooley C. M. Digby Z. (2017). A high-resolution mRNA expression time course of embryonic development in zebrafish. Elife 6, e30860. 10.7554/eLife.30860 29144233 PMC5690287

[B88] Windner S. E. Doris R. A. Ferguson C. M. Nelson A. C. Valentin G. Tan H. (2015). Tbx6, Mesp-b and Ripply1 regulate the onset of skeletal myogenesis in zebrafish. Development 142, 1159–1168. 10.1242/dev.113431 25725067 PMC4360180

[B89] Xing C. Shen W. Gong B. Li Y. Yan L. Meng A. (2022). Maternal factors and nodal autoregulation orchestrate nodal gene expression for embryonic mesendoderm induction in the zebrafish. Front. Cell Dev. Biol. 10, 887987. 10.3389/fcell.2022.887987 35693948 PMC9178097

[B90] Xu P. Zhu G. Wang Y. Sun J. Liu X. Chen Y. G. (2014). Maternal Eomesodermin regulates zygotic nodal gene expression for mesendoderm induction in zebrafish embryos. J. Mol. Cell Biol. 6, 272–285. 10.1093/jmcb/mju028 24924767

[B91] Yates A. Akanni W. Amode M. R. Barrell D. Billis K. Carvalho-Silva D. (2016). Ensembl 2016. Nucleic Acids Res. 44, D710–D716. 10.1093/nar/gkv1157 26687719 PMC4702834

[B92] Yu X. Ng C. P. Habacher H. Roy S. (2008). Foxj1 transcription factors are master regulators of the motile ciliogenic program. Nat. Genet. 40, 1445–1453. 10.1038/ng.263 19011630

