# Supplementary material for: Eomes function is conserved between zebrafish and mouse and controls left-right organiser progenitor gene expression via interlocking feedforward loops
Source: Front Cell Dev Biol. 2022 Aug 25;10:982477. doi: 10.3389/fcell.2022.982477 (PMC9483813; doi:10.3389/fcell.2022.982477)
Supplement: Supplementary file 2 [file Image1.pdf]

Supplementary Material

Supplementary Figures

Figure S1

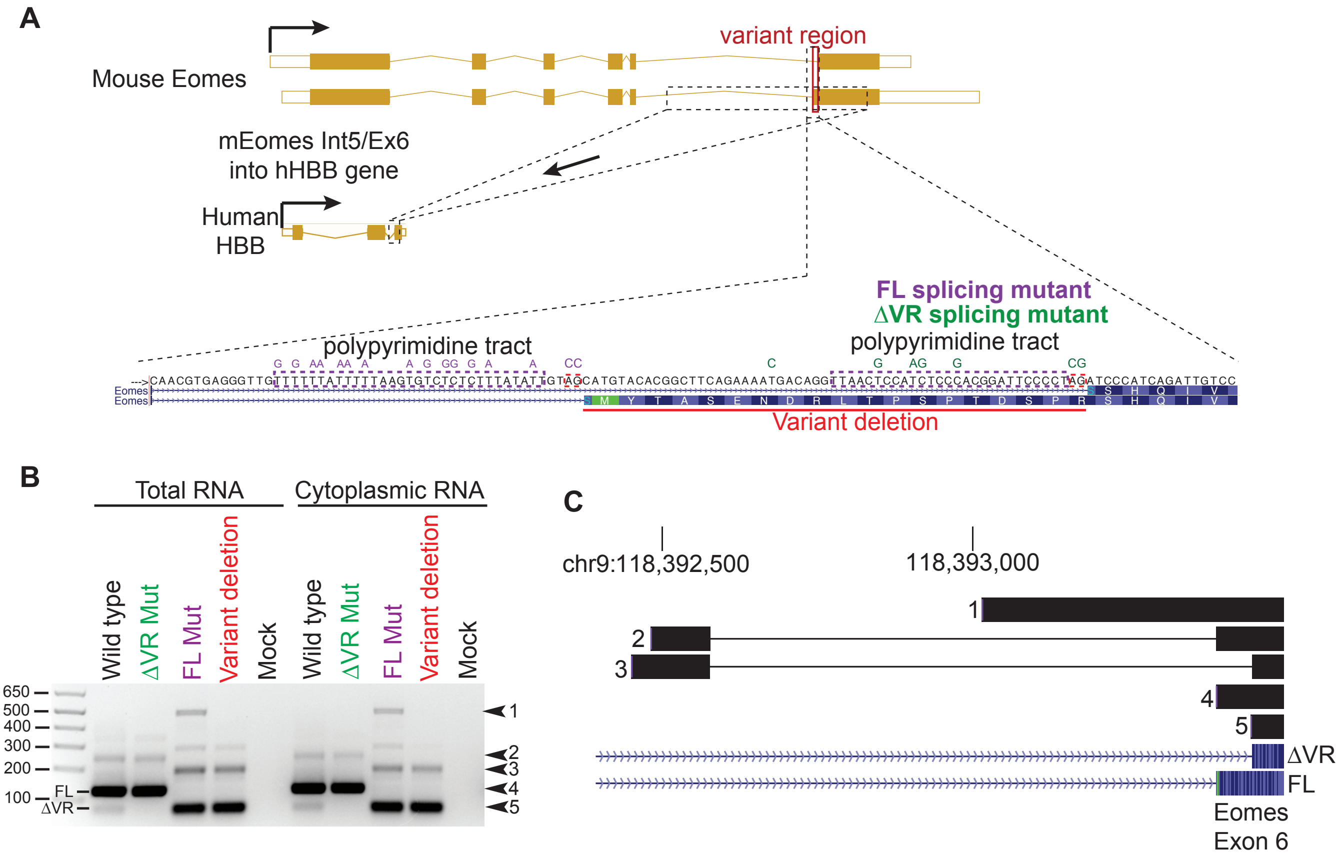

Figure S1. Mutational analysis of exon 6 splicing sequences indicate that the *Eomes* ΔVR splice site is weak compared to the *Eomes* FL splice site.

(A) Schematic of hybrid gene construction and mutagenesis to investigate FL and ΔVR exon 6 splicing consensus sequences. *Eomes* intron5/exon 6 sequences cloning substitution into the body of *HBB* is indicated. Base mutations or deletions in each construct are colour coded. All mutations within the VR are synonymous. Splice acceptor sequences are in dashed red boxes. Mutations indicated in purple are intended to prevent splicing into the FL splice acceptor, both by eliminating the AG splice acceptor itself and replacing pyrimidines with purines in the polypyrimidine tract. The mutations in green are similarly intended to prevent splicing into the ΔVR splice acceptor. The region deleted in the variant deletion is underlined in red.

(B) RT-PCR revealing HBB exon 2/Eomes exon 6 splicing events in HBB/Eomes hybrid genes. Arrows indicate sequenced PCR products. Note that the ΔVR splicing event is uncommon, reflecting other results. Further note that mutation of ΔVR splicing sequences leads to all splicing into the FL site, while mutation of the FL splicing sequences leads to discovery of intron 5 cassette exons rather than complete splicing into ΔVR, potentially reflecting weakness of ΔVR splicing sequences.

(C) Genomic alignments of RT-PCR products labelled in panel B. Sequences of RT-PCR products obtained by Sanger sequencing are in Table S1.

Figure S2

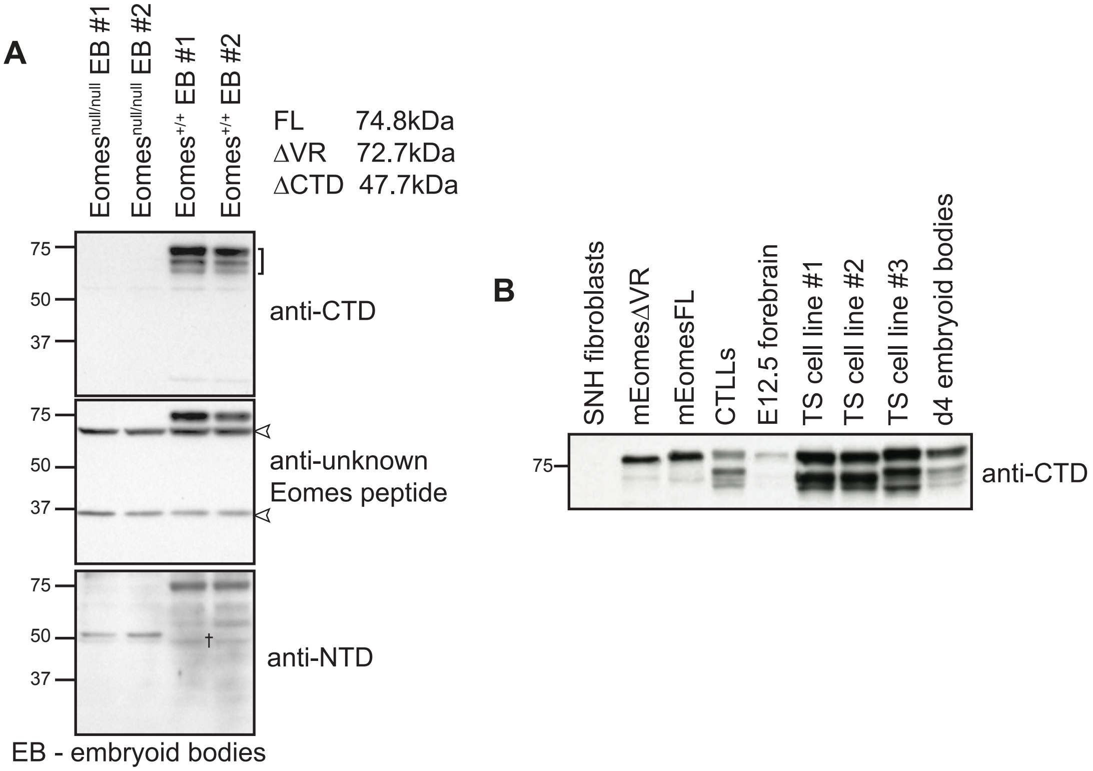

Figure S2. Analysis of *Eomes* protein products indicates lack of ΔCTD isoform and multiple N-terminal deletions.

(A) Western blot analysis of Eomes null and wild type day 4 embryoid bodies using antibodies recognizing different regions of the Eomes protein. Bracket indicates Eomes bands identified by anti-CTD antibody. Open arrowheads indicate non-specific bands. † indicates potential ΔCTD isoform band.

(B) Western blot analysis of different Eomes expressing cell types. SNH fibroblasts are to control for background signal.

Figure S3

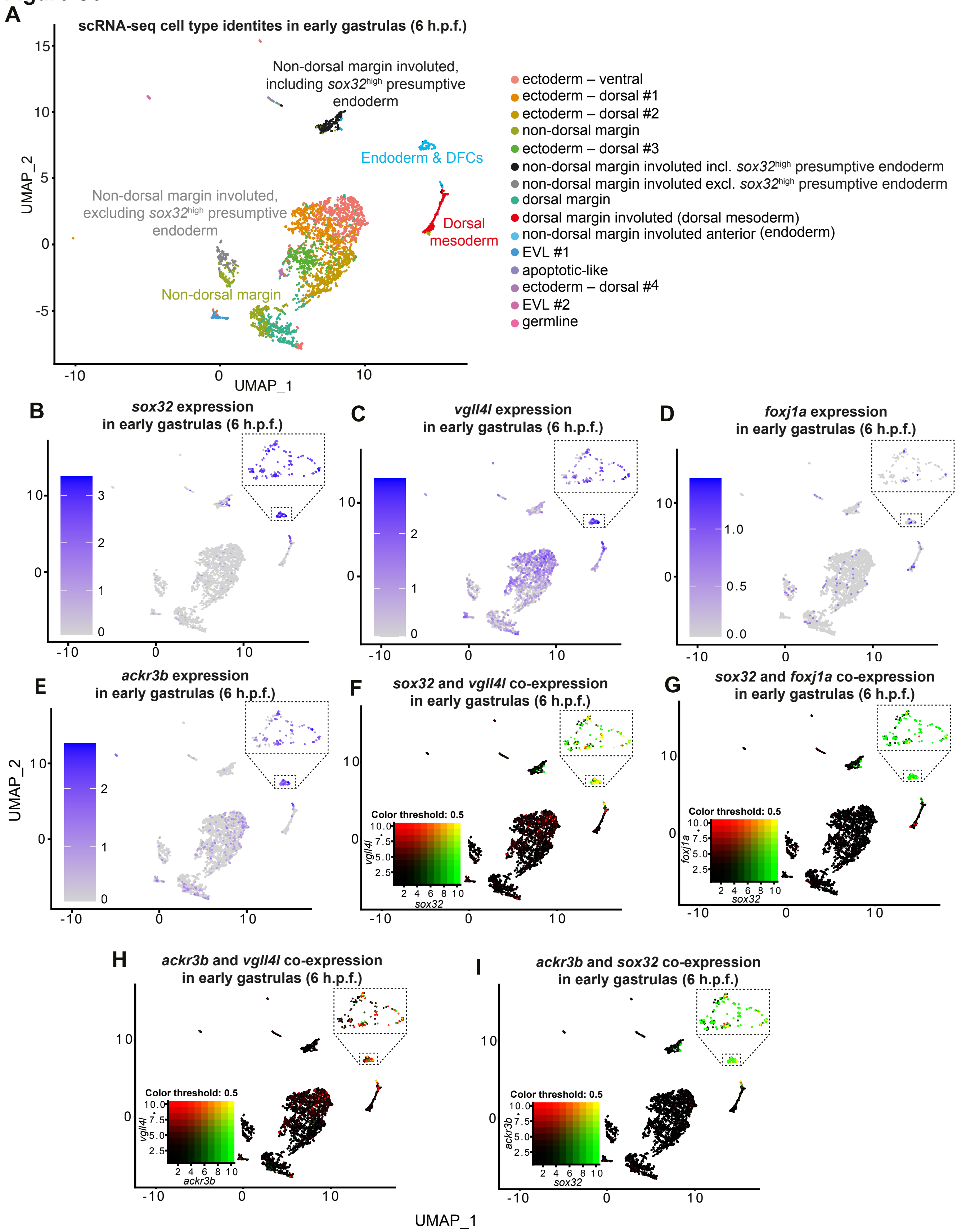
A
scRNA-seq cell type identites in early gastrulas (6 h.p.f.)
Non-dorsal margin involuted, including sox32high presumptive endoderm
Endoderm & DFCs
Non-dorsal margin involuted, excluding sox32high presumptive endoderm
Dorsal mesoderm
Non-dorsal margin
UMAP_1
UMAP_2
ectoderm – ventral
ectoderm – dorsal #1
ectoderm – dorsal #2
non-dorsal margin
ectoderm – dorsal #3
non-dorsal margin involuted incl. sox32high presumptive endoderm
non-dorsal margin involuted excl. sox32high presumptive endoderm
dorsal margin
dorsal margin involuted (dorsal mesoderm)
non-dorsal margin involuted anterior (endoderm)
EVL #1
apoptotic-like
ectoderm – dorsal #4
EVL #2
germline
B
sox32 expression in early gastrulas (6 h.p.f.)
C
vgll4l expression in early gastrulas (6 h.p.f.)
D
foxj1a expression in early gastrulas (6 h.p.f.)
E
ackr3b expression in early gastrulas (6 h.p.f.)
F
sox32 and vgll4l co-expression in early gastrulas (6 h.p.f.)
G
sox32 and foxj1a co-expression in early gastrulas (6 h.p.f.)
H
ackr3b and vgll4l co-expression in early gastrulas (6 h.p.f.)
I
ackr3b and sox32 co-expression in early gastrulas (6 h.p.f.)
Color threshold: 0.5
UMAP_1

Figure S3. Identities of cell clusters within early gastrula (6 h.p.f.) scRNA-seq data.

(A) UMAP clustering analysis of single-cell RNA-seq data from Wagner *et al.* (2018) for early gastrula (6 h.p.f.) zebrafish embryos indicating the predicted cell type identities. The identities in the key use the same terminology defined by Wagner *et al.* For consistency, identities were assigned by identifying transcripts enriched in each cluster, and comparing to the markers for each cluster defined by Wagner *et al.* "Non-dorsal margin involuted" gives rise to both endoderm and mesoderm derivatives and as such the "non-dorsal margin involuted" cluster containing *sox32*-expressing cells contains the presumptive endoderm. Markers used to assign cluster identities are in Table S2.

(B) UMAP clustering as panel A indicating expression of endoderm and DFC marker *sox32*. Heatmap inset represents log normalized gene expression level.

(C) As panel B indicating expression of DFC marker *vgll4l*. Note that *vgll4l* is also known to be expressed in ventral ectoderm.

(D) As panel B indicating expression of DFC marker *foxj1a*.

(E) As panel B indicating expression of endoderm marker *ackr3b*.

(F) UMAP clustering analysis of single-cell RNA-seq data for early gastrula (6 h.p.f.) zebrafish embryos indicating co-expression of *sox32* and *vgll4l* (Wagner et al., 2018). Heatmap insets indicate overall expression levels per gene and co-expression. Overlapping expression is shown in yellow. *Sox32* is expressed by endoderm and DFCs, while *vgll4l* is considered to be a DFC marker. This suggests that endoderm and DFCs cannot be clearly resolved as separate clusters at this stage of development with the number of cells available within the dataset.

(G) As panel F, indicating co-expression of *sox32* and *foxj1a*. *Foxj1a* is a DFC marker, again suggesting that endoderm and DFCs are part of the same cluster in this dataset.

(H) As panel F, indicating co-expression of *ackr3b* and *vgll4l*. *Ackr3b* is and endoderm marker, suggesting distinction between endoderm and DFC cells.

(I) As panel F, indicating co-expression of *ackr3b* and *sox32*.

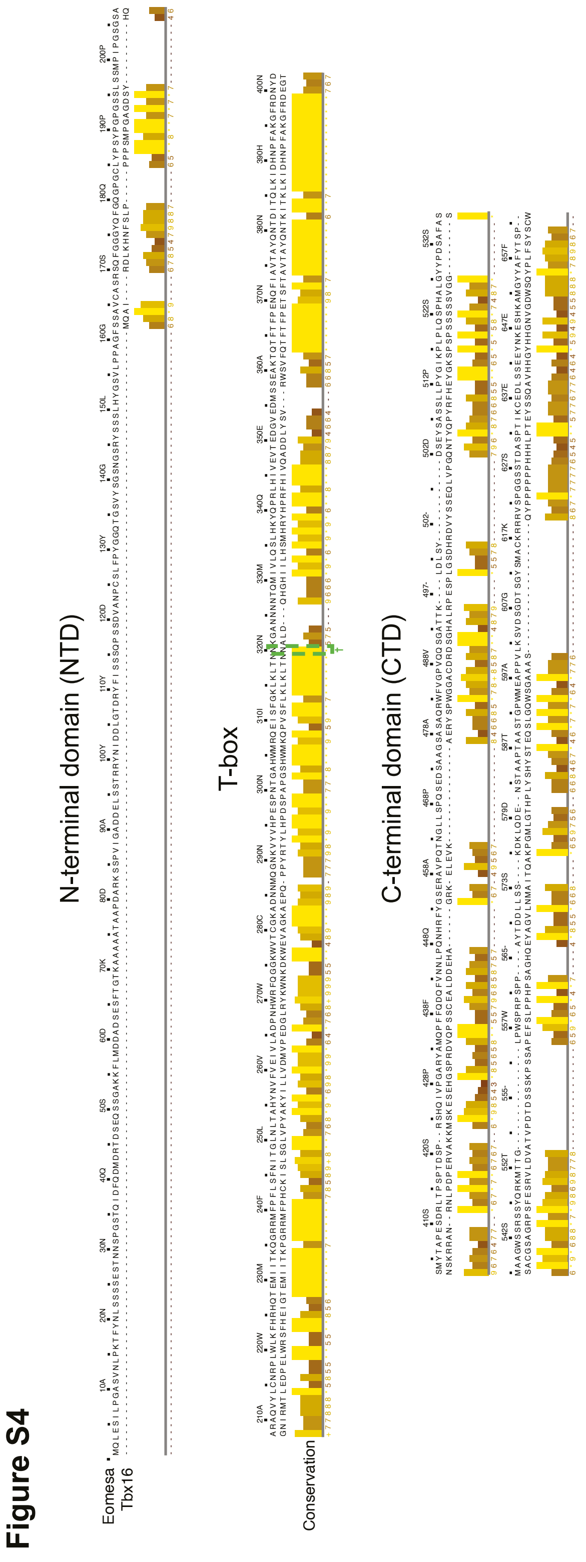

Figure S4. Jalview alignment of zebrafish Eomesa and Tbx16 proteins separated by domain. The conserved N amino acid at Eomesa position 320 is outlined in green. Conservation scores indicate similarity of physico-chemical properties of aligned amino acids

Figure S5

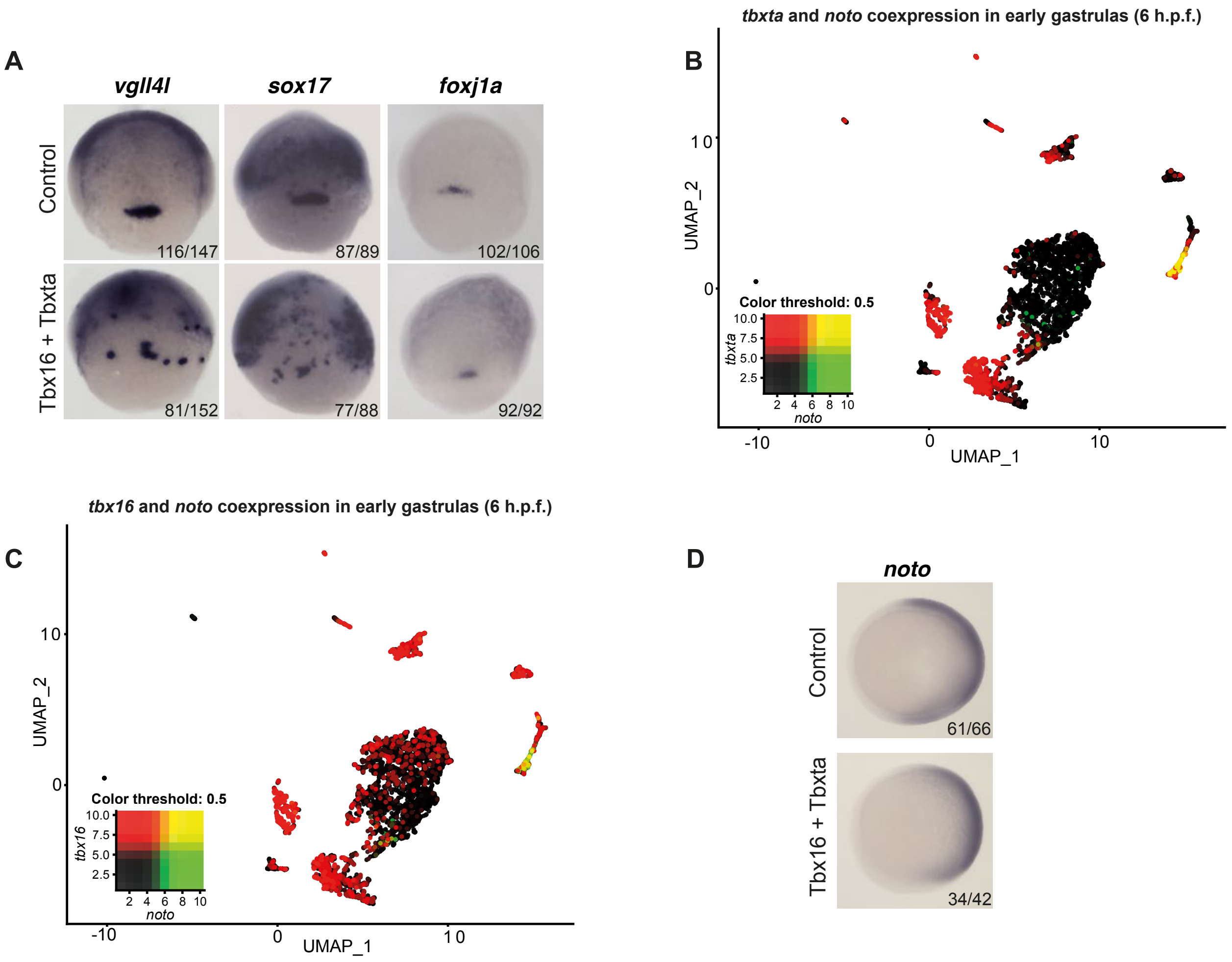

Figure S5. Combinatorial overexpression of Tbx16 and Tbxta leads to dispersion of a subset of DFC markers, but not an overall expansion of dorsal markers.

(A) Dorsal views of control or Tbx16 + Tbxta overexpressing early/mid gastrulas (6 h.p.f.). Fraction of embryos exhibiting the displayed *vgll4l*, *sox17* and *foxj1a* WISH expression patterns are indicated.

(B) UMAP clustering analysis of single-cell RNA-seq data for early gastrulas (6 h.p.f.) zebrafish embryos indicating co-expression of *tbxta* and *noto* (Wagner et al., 2018). Heatmap insets indicate overall expression levels per gene and co-expression. Overlapping expression is shown in yellow.

(C) As panel B, indicating co-expression of *tbx16* and *noto*.

(D) Animal pole views of control or Tbx16 + Tbxta overexpressing early/mid gastrulas (6 h.p.f.) exhibiting *noto* WISH expression patterns. Fraction of embryos exhibiting the displayed expression patterns are indicated.
